# Analytical protocols for Phobos regolith samples returned by the Martian Moons eXploration (MMX) mission

**DOI:** 10.1186/s40623-021-01438-9

**Published:** 2021-06-01

**Authors:** Wataru Fujiya, Yoshihiro Furukawa, Haruna Sugahara, Mizuho Koike, Ken-ichi Bajo, Nancy L. Chabot, Yayoi N. Miura, Frederic Moynier, Sara S. Russell, Shogo Tachibana, Yoshinori Takano, Tomohiro Usui, Michael E. Zolensky

**Affiliations:** 1 Ibaraki University, 2-1-1 Bunkyo, Mito, Ibaraki 310-8512 Japan; 2 Tohoku University, 6-3 Aza-aoba, Aramaki, Aoba-ku, Sendai, 980-8578 Japan; 3 Institute of Space and Astronautical Science, JAXA, 3-1-1 Yoshinodai, Sagamihara, Kanagawa 252-5210 Japan; 4 Hiroshima University, 1-3-1 Kagamiyama, Higashihiroshima, Hiroshima 739-8526 Japan; 5 Department of Earth and Planetary Sciences, Hokkaido University, N10W8 Kita-ku, Sapporo, 060-0810 Japan; 6 Johns Hopkins University Applied Physics Laboratory, 11100 Johns Hopkins Rd, Laurel, MD 20723 USA; 7 Earthquake Research Institute, University of Tokyo, 1-1-1 Yayoi, Bunkyo-ku, Tokyo, 113-0032 Japan; 8 Institut de Physique du Globe de Paris, CNRS, University of Paris, Paris, France; 9 Department of Earth Sciences, Natural History Museum, Cromwell Road, London, SW7 5BD UK; 10 UTOPS, University of Tokyo, 7-3-1 Hongo, Bunkyo-ku, Tokyo, 113-0033 Japan; 11 Biogeochemistry Research Center, Japan Agency for Marine-Earth Science and Technology, 2-15 Natsushima, Yokosuka, 237-0061 Japan; 12 ARES, NASA Johnson Space Center, Houston, TX 77058 USA

**Keywords:** MMX, Sample analyses, Mineralogy, Petrology, Chemical composition, Isotopic composition, Organic matter

## Abstract

Japan Aerospace Exploration Agency (JAXA) will launch a spacecraft in 2024 for a sample return mission from Phobos (Martian Moons eXploration: MMX). Touchdown operations are planned to be performed twice at different landing sites on the Phobos surface to collect > 10 g of the Phobos surface materials with coring and pneumatic sampling systems on board. The Sample Analysis Working Team (SAWT) of MMX is now designing analytical protocols of the returned Phobos samples to shed light on the origin of the Martian moons as well as the evolution of the Mars–moon system. Observations of petrology and mineralogy, and measurements of bulk chemical compositions and stable isotopic ratios of, e.g., O, Cr, Ti, and Zn can provide crucial information about the origin of Phobos. If Phobos is a captured asteroid composed of primitive chondritic materials, as inferred from its reflectance spectra, geochemical data including the nature of organic matter as well as bulk H and N isotopic compositions characterize the volatile materials in the samples and constrain the type of the captured asteroid. Cosmogenic and solar wind components, most pronounced in noble gas isotopic compositions, can reveal surface processes on Phobos. Long- and short-lived radionuclide chronometry such as ^53^Mn–^53^Cr and ^87^Rb–^87^Sr systematics can date pivotal events like impacts, thermal metamorphism, and aqueous alteration on Phobos. It should be noted that the Phobos regolith is expected to contain a small amount of materials delivered from Mars, which may be physically and chemically different from any Martian meteorites in our collection and thus are particularly precious. The analysis plan will be designed to detect such Martian materials, if any, from the returned samples dominated by the endogenous Phobos materials in curation procedures at JAXA before they are processed for further analyses.

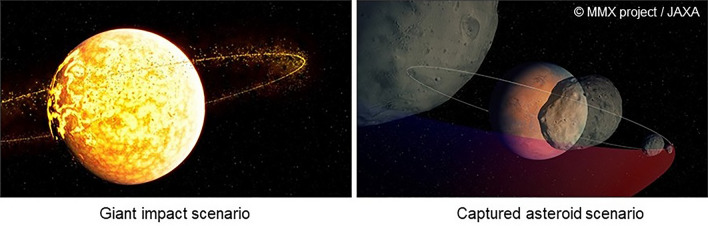

## Martian Moons eXploration (MMX)

How and when terrestrial planets acquired volatiles has been a long-standing debate (e.g., Dreibus and Wanke 1985; Albarède 2009; Marty 2012). Especially, the source of water, e.g., the solar nebula, planetesimals/asteroids, or comets, has been of considerable interest since it has played important roles in shaping the surface environment, controlling climate stability, and supporting the emergence and evolution of life (e.g., Genda 2016). Although Mars shows several lines of evidence for the past presence of liquid water as well as for a thick atmosphere compared with the present inferred from valley formation, erosion, hydrous weathering, and a mineral record of water–rock interactions, these are not the characteristics of Mars today (e.g., Carr and Head 2010; Ehlmann et al. 2016; Usui 2019). The origin of the Martian water and atmosphere and the mechanisms of their loss over time would provide a better understanding of the surface evolution and climate change of terrestrial planets (Usui et al. 2012, 2015; Kurokawa et al. 2015; Deng et al. 2020; Jakosky 2021). Moreover, Mars has two small and irregular-shaped satellites: Phobos and Deimos. The Martian moons must have witnessed the formation processes of the Mars–moon system and the volatile delivery to the planet. Although the origin of the Martian moons still remains unknown (Rosenblatt 2011), it is closely related to the surface evolution of Mars and the acquisition of its volatiles.

Japan Aerospace Exploration Agency (JAXA) will launch a spacecraft in 2024 for a sample return mission from Phobos: Martian Moons eXploration (MMX). Touchdown operations will be performed to collect > 10 g of the Phobos surface materials. The scientific goals of the MMX mission are (i) to reveal the origin of Martian moons, and to make progress in our understanding of planetary system formation, and of primordial material transport around the border between the inner and outer regions of the early solar system, and (ii) to observe processes that impact the evolution of the Mars system from a new vantage point, and to advance our understanding of Mars’ surface environment transition (Kuramoto et al. 2018; Usui et al. 2020) (Table 1). Analyses of the Phobos regolith samples returned by MMX along with remote sensing observations of the Phobos surface are expected to place key constraints on the origin of the Martian moons and the evolution of the Mars–moon system. Note that the National Academies of Sciences, Engineering, and Medicine (2019) has recommended that missions returning samples from the Martian moons should be classified as “unrestricted” Earth return in the framework of the planetary protection policy maintained by the International Council for Scientific Unions (ICSU) Committee on Space Research (COSPAR).

**Table 1:** Mission goals and objectives, and key sample analyses

MMX goals	MMX objectives	Key sample analyses to address objectives
Goal 1. To reveal the origin of the Mars moons, and then to make progress in our understanding of planetary system formation and of primordial material transport around the border between the inner- and the outer-part of the early solar system	Objective 1.1. To determine whether the origin of Phobos is captured asteroid or giant impact	Texture and mineral composition; major element composition; O- and Cr-isotopes
	Objective 1.2a. (In the case of captured asteroid origin) To understand the primordial material delivery process to the rocky planet region and to constrain the initial condition of the Mars surface environment	O- and Cr-isotopes; texture and mineral composition; major element composition; volatile/refractory elements; H-, C-, and N-isotopes; organic matter; presolar grains; remnant magnetization; K–Ar systematics; Al–Mg and Mn–Cr systematics; U–Pb systematics
	Objective 1.2b. (In the case of giant impact origin) To understand the satellite formation via giant impact and to evaluate how the initial evolution of the Mars environment was affected by the moon forming event	O- and Cr-isotopes; texture and mineral composition; major element compositions; isotopes of moderately volatile elements; siderophile elements; K–Ar systematics; U–Pb systematics; Rb–Sr systematics
	Objective 1.3. To constrain the origin of Deimos	Texture and mineral composition; major element composition; O- and Cr-isotopes
Goal 2. To observe processes that have impact on the evolution of the Mars system from the new vantage point and to advance our understanding of Mars surface environment transition	Objective 2.1. To obtain a basic picture of surface processes of the airless small body in its orbit around Mars	Space-weathering layer; solar wind and cosmogenic components; micrometeorite bombardment; K–Ar systematics; U–Pb systematics
	Objective 2.2. To gain new insight on Mars' surface environment evolution	O- and Cr-isotopes; texture and mineral compositions; major element compositions; K–Ar systematics; U–Pb systematics; remnant magnetization; H-isotopes

Regolith samples on Phobos are planned to be collected at two different landing sites. Phobos’ surface exhibits heterogeneous reflectance spectra consisting of two fundamental spectral units, ‘redder unit’ and ‘bluer unit’. The spectra of the two units are both dark, and the ‘redder unit’ shows increasing reflectance with increasing wavelength, whereas the ‘bluer unit’, which makes up the interior and ejecta of the large crater Stickney, has a somewhat flatter spectrum (e.g., Murchie and Erard 1996). Only the redder unit can be well compared with other low-albedo bodies (C- and D-type asteroids), but it is redder than and distinct from them. If Phobos regolith samples are collected from both spectral units, then the representativeness of the returned samples and the mechanism to cause such a spectral variation could be elucidated.

Two independent sampling systems using coring (C-) and pneumatic (P-) samplers will be employed in the MMX mission (Usui et al., 2020). The C-sampler is operated using two core soil tubes with another backup tube, each of which can collect > 10 g of regolith samples (Kato et al. 2020). These coring tubes can penetrate the Phobos regolith and collect samples deeper than 2 cm. Penetration, vibration, and other tests on Phobos soil simulants conducted by JAXA will evaluate to what extent the stratigraphy of the Phobos surface materials can be preserved. On the other hand, the P-sampler will collect regolith samples at the very surface of Phobos (Zacny et al. 2020). Thus, the regolith samples collected by the P-sampler may show pronounced influence of solar wind, cosmic rays, and micrometeorite bombardment, like the regolith samples on the asteroid Itokawa returned by the Hayabusa spacecraft (Nagao et al. 2011; Noguchi et al. 2011).

The Sample Analysis Working Team (SAWT) of MMX is now designing analytical protocols of the returned Phobos samples. The analytical protocols are aiming at maximizing the scientific results to be obtained from the returned samples. In this paper, we first summarize the proposed formation scenarios of the Martian moons and the expected characteristics of the samples returned by MMX. Next, we show the initial analysis plans, separately designed for inorganic and organic analyses, to achieve the scientific objectives of MMX. Then, we consider a special case where materials possibly transported from Mars to Phobos are detected in the returned samples. Finally, we mention the preliminary examination of the returned samples to be carried out together with curation procedures.

## Formation scenarios of the Martian moons and the expected characteristics of samples returned by MMX

The origin of the Martian moons is controversial. Currently favored formation scenarios of the Martian moons include (i) the captured asteroid scenario, where asteroids originating outside the Mars system were gravitationally captured when passing by Mars (e.g., Hartmann 1990; Higuchi and Ida 2017), and (ii) the in situ formation scenario, where the moons formed from a debris disk surrounding Mars produced by a giant impact after the formation of Mars (e.g., Citron et al. 2015; Rosenblatt et al. 2016; Hyodo et al. 2017). It is also possible that these moons formed by different mechanisms, though they share similar traits, such as irregular shapes and spectral features.

### In the case of the captured asteroid scenario

The captured asteroid hypothesis is supported by the spectral features from visible to near-infrared wavelength of Phobos and Deimos resembling D- or T-type asteroids possibly with indigenous water and organic-rich features (e.g., Murchie and Erard 1996; Rivkin et al. 2002a; Kanno et al. 2003; Fraeman et al. 2012, 2014; Pajola et al. 2013; Yamamoto et al. 2018). Unique and primitive carbonaceous chondrites Tagish Lake and Wisconsin Range (WIS) 91600 rich in organic matter (e.g., Pizzarello et al. 2001; Nakamura-Messenger et al. 2006; Herd et al. 2011) show spectral similarities to D- or T-type asteroids (Hiroi et al. 2001, 2005). Although D- and T-type asteroids are currently located in the outer regions of the main asteroid belt and in the Jupiter Trojan regions (DeMeo and Carry 2014), they may have formed in the distal solar system (e.g., Vokrouhlický et al. 2016; Fujiya et al. 2019; Bryson et al. 2020). The low density of Phobos is also similar to that of Tagish Lake, suggesting their nature as a primitive, porous material (Brown et al. 2000; Pätzold et al. 2014). It is also possible that the captured small body was a comet or an extinct comet. In the case of the captured asteroid scenario, like primitive carbonaceous chondrites of outer solar system origins, the moons are predicted to contain crystalline silicates including phyllosilicates, carbonates, oxides, organic matter, and possibly, amorphous silicates (Zolensky et al. 2002; Brearley 2006; Nakamura-Messenger et al. 2006; Alexander et al. 2007; Leroux et al. 2015; Krot et al. 2015). The Rosetta mission led by ESA revealed that the comet 67P/Churyumov Gerasimenko has low reflectance spectra, due to the presence of dark refractory polyaromatic carbonaceous materials with opaque mineral phases (Quirico et al. 2016). The in situ analysis of the cometary dust particles by a mass spectrometer on board (The COmetary Secondary Ion Mass Analyzer: COSIMA) also revealed that they are composed of abundant organic matter comprising nearly 45% in mass with mostly anhydrous minerals (Bardyn et al. 2017). Carbonaceous chondrites contain many types of organic compounds including volatile and semi-volatile organic matter, soluble organic matter (SOM), and solvent-insoluble organic matter (IOM) that formed before and after the accretion of their parent asteroids (e.g., Sephton et al. 2003; Derenne and Robert 2010). Comets and primitive carbonaceous chondrites of outer solar system origins are expected to contain more labile organic compounds (i.e., amino acids, sugars, carboxylic acids, and short-chain hydrocarbons).

The inner solar system origins of captured asteroids cannot be ruled out, and in that case, the moons may consist of volatile-poor chondritic materials with reduced, anhydrous materials such as olivine, pyroxene, Fe–Ni metal, Fe–Ni sulfides, all of which can be found in ordinary chondrites (Brearley and Jones 1998). Refractory inclusions and chondrules, which characterize primitive chondrites, may also be contained by the moons (Scott and Krot 2003). An argument against the captured asteroid hypothesis is that it is challenging to explain the current orbital parameters of Phobos and Deimos, i.e., their near-circular and near-equatorial orbits (e.g., Burns 1992; Rosenblatt 2011).

### In the case of the giant impact scenario

Instead, the current orbits of the Martian moons favor the giant impact scenario. From crater chronology, the formation or major collision of Phobos would have occurred 4.3 Gyr ago if Phobos has been in its current orbit about Mars since its formation (Schmedemann et al. 2014). In that scenario the low density of Phobos can be explained by a significant amount of porosity resulting from piling up impact debris. A numerical simulation suggests that a giant impact capable of creating Borealis basin, a large basin in the northern hemisphere of Mars, could disperse sufficient amounts of debris from which at least one of the moons could have formed (Citron et al. 2015). A recently proposed scenario is that Martian satellites accreted within a debris disk, and an outward migration of larger satellites may have facilitated accretion of two smaller satellites which eventually evolved into Phobos and Deimos, while the larger satellites, in turn, fell back to Mars due to its tidal pull (Rosenblatt et al. 2016).

The disk materials, i.e., the building blocks of the Martian moons, were likely heated to temperatures as high as 2000 K (Hyodo et al. 2017; Canup and Salmon 2018), and thus, the moons may consist of glassy or recrystallized igneous materials such as olivine, pyroxene, and plagioclase. At such high temperatures, most volatile materials (e.g., water and organic materials) would have been lost. Thus, organic materials on Phobos, if present, would have been derived by meteorites and/or from Mars after the formation of Phobos. In the case of the giant impact scenario, the ingredients of the Martian moons are predicted to be a mixture of both Martian and impactor materials with a mixing ratio depending on the impact angle (Hyodo et al. 2017). The nature of the impactor is difficult to predict but could be constrained by comparing the composition of returned Phobos regolith samples to the composition expected from condensation of a gas and solids from a cooling melt in the impact-generated disk (Pignatale et al. 2018), and composition and mineralogy of known Martian meteorites. Because the Martian materials contributing to the Martian moons would originate from regions as deep as ~ 100 km from the Martian surface (Hyodo et al. 2017), the moons could contain both Martian crust and mantle materials but would be depleted in metals with siderophile elements that had already formed the Martian core (Righter and Chabot 2011).

### Exogenous materials

It should be noted that materials ejected from Mars to Phobos by asteroidal impacts might account for a few hundreds of ppm of the entire Phobos regolith (Ramsley and Head 2013; Hyodo et al. 2019). The asteroidal impact events associated with the delivery of Martian materials to Phobos may have lasted throughout Martian history. Therefore, the materials ejected from Mars might provide unique information about the surface evolution of Mars, whereas crystallization ages of most Martian meteorites are younger than 1.3 Ga (Nyquist et al. 2001).

Since the Martian materials ejected to Phobos are likely located near the surface of Mars, some of them should contain upper crustal materials found in Martian meteorites such as shergottites (McSween 2015). However, a numerical simulation shows that the impact ejecta from Mars to Phobos would also include physically and chemically different materials from the Martian meteorites, because the former possibly includes less-shocked materials than the latter which is commonly shocked by > 5 GPa to eject them to Earth (Fritz et al. 2005; Hyodo et al. 2019). Therefore, sedimentary rocks, which have widely been observed on Mars but have rarely been found in Martian meteorites likely due to their fragile nature and destruction during the ejection impact, could be delivered to Phobos (e.g., Malin and Edgett 2000; Lewis et al. 2008). Clays, carbonates, sulfates, and chlorides are included in Martian sediments (Ehlmann and Edwards 2014), and could also be present in Phobos’ regolith. Thus, if Martian sedimentary rocks can be detected in the returned samples, they would provide crucial information about the surface evolution of Mars, such as eolian erosion and aqueous alteration, which cannot be obtained from Martian meteorites. Perhaps, we will see even biomarkers inherited from Mars, although MMX has been classified as “unrestricted” Earth Return without any concern about viable Phobos organisms to be returned.

Deimos materials ejected by impacts could also be transferred to Phobos and the time-averaged flux of Deimos dust would be as high as 11% of the direct impactor flux on Phobos (Nayak et al. 2016). It would be difficult to distinguish between Phobos original materials and Deimos dust transferred to Phobos if the two moons have a similar composition, such as predicted in the giant impact scenario for the Martian moons’ origin. If Phobos and Deimos are captured asteroids of different types, Deimos materials could potentially be identified in the returned samples by having distinct compositional and isotopic signatures, such as seen in different meteorite types. Remote sensing observations indicate that there is spectral similarity between Deimos and Phobos’ “redder unit” (Fraeman et al. 2012), and thus if MMX samples are obtained from both of Phobos’ spectral units, the returned samples are well suited to search for potential components from Deimos as well.

## Key analyses of returned samples and connections to remote sensing observations

**Fig. 1:**
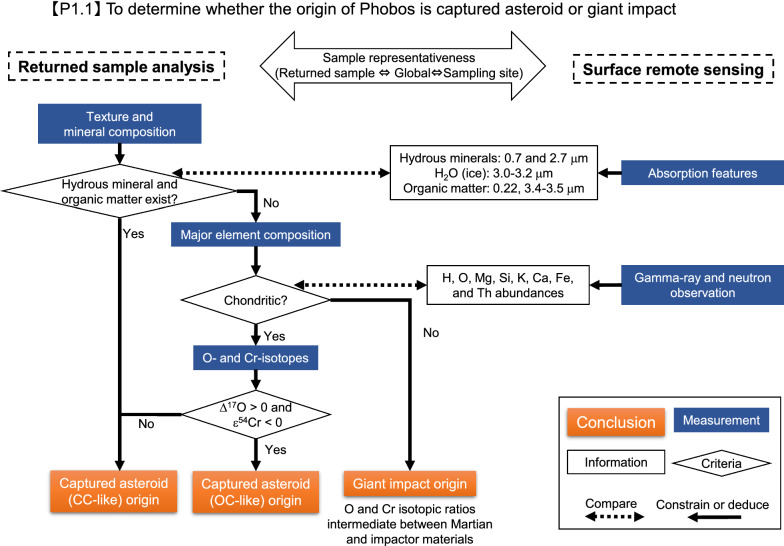
Flowchart of sample analyses together with remote sensing observations to determine the origin of Phobos (objective 1.1 in Table 1)

**Fig. 2:**
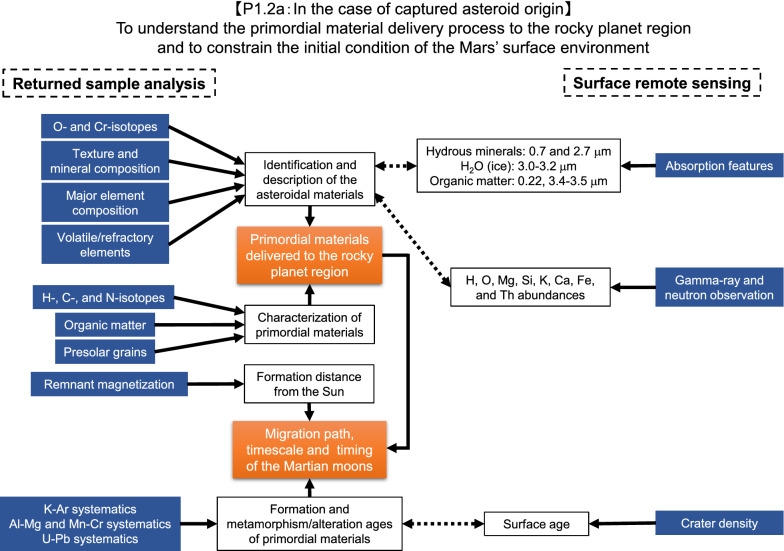
Flowchart of sample analyses together with remote sensing observations to characterize the Phobos building blocks in the case of the captured asteroid origin (objective 1.2a in Table 1)

**Fig. 3:**
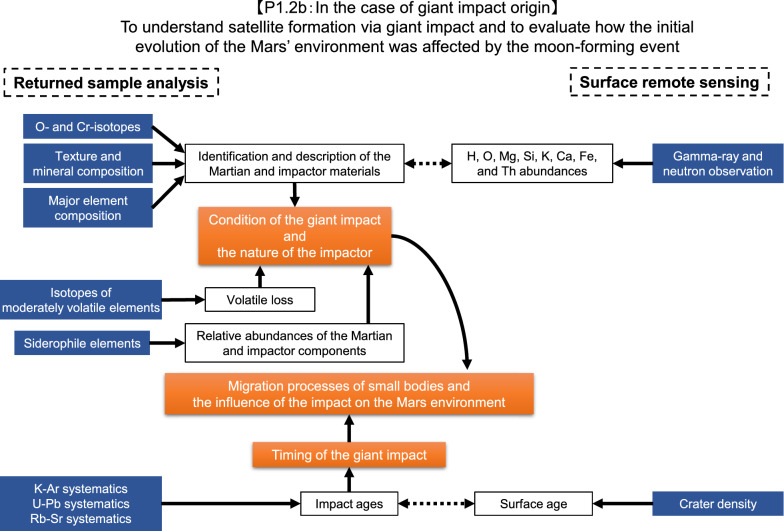
Flowchart of sample analyses together with remote sensing observations to characterize the Phobos building blocks in the case of the giant impact origin (objective 1.2b in Table 1)

**Fig. 4:**
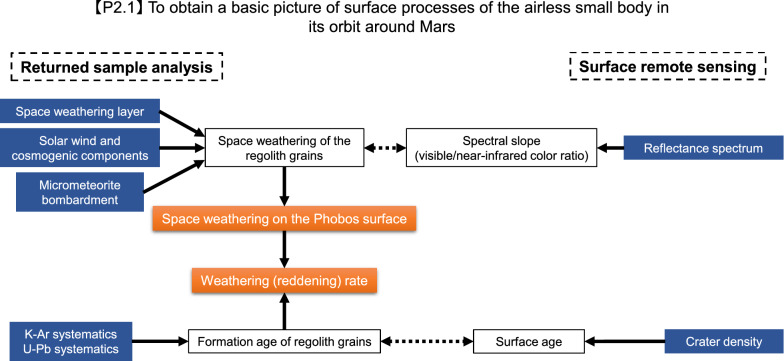
Flowchart of sample analyses together with remote sensing observations to investigate the surface processes on Phobos (objective 2.1 in Table 1)

Key analyses of the returned Phobos samples are summarized by Usui et al. (2020). As described below, the key analyses and remote sensing observations are complementary (Figs. 1, 2, 3, 4) and designed to work together to achieve the mission objectives (Table 1).

### To determine the origin of Phobos

Valuable information about the origin of the Phobos building blocks can be derived from mineralogical and petrological observations, and a micro-analysis of the physical properties of the returned samples (Fig. 1). The physical properties such as density, porosity, and strength of the returned samples can be compared to macroscopic measurements of Phobos from remote sensing observations. In the case of the captured asteroid scenario, the mineralogical and petrological information of the returned samples will permit direct comparison between them and known meteorite groups such as CM, CI, and CR chondrites as well as the Tagish Lake meteorite (e.g., Bunch and Chang 1980; Tomeoka and Buseck 1988; Weisberg et al. 1993; Zolensky et al. 2002). Based on the matrix mineralogy of the samples, their primitiveness and their degrees of aqueous alteration can be evaluated (Noguchi et al. 2017). In the case of the giant impact scenario, the mineralogy and petrology of the returned samples can be compared with those expected for condensation from gas and solids from a cooling melt in the impact-generated disk, calculated assuming thermodynamic equilibrium (Pignatale et al. 2018). The mineralogy and petrology of the samples can be corroborated by remote sensing observations of the Phobos surface (e.g., the presence/absence of absorption at ~ 0.7 and ~ 2.7 μm indicative of hydrated minerals and at ~ 3.4 μm for macromolecular organic solids; Rivkin et al. 2002b). Moreover, it is important to investigate possible difference in the mineralogy/petrology of the samples from two sampling sites, especially between the ‘redder’ or ‘bluer’ spectral units.

Chemical compositions of the returned samples can be used to distinguish between chondritic and Martian/impactor materials. The chemical composition of the Phobos surface measured by remote sensing observations, such as by the gamma-ray and neutron spectrometer measurements of MMX’s MEGANE (Mars–Moon Exploration with Gamma rays and Neutrons) instrument (Lawrence et al. 2019), can be compared with those of the returned samples, to provide global compositional context for interpretation of the samples. In addition, the compositions of Martian igneous rocks analyzed by rovers and of Martian meteorites can be utilized as references if the returned samples show diagnostic characteristic in favor of the giant impact scenario (McSween 2015).

Non-mass-dependent isotopic compositions of, e.g., O, Ca, Ti, and Cr are a powerful tool to characterize the source materials from which the samples formed and possibly their locations. Different meteorite groups, each of which is generally considered to have derived from distinct types of asteroids, show unique isotopic signatures for these elements (e.g., Clayton 1993; Trinquier et al. 2007, 2009; Schiller et al. 2018). Especially, differences between carbonaceous chondrites (CCs) and non-carbonaceous chondrites (NCs), the so-called isotopic dichotomy, is pronounced in a plot of Δ^17^O against ε^54^Cr or ε^50^Ti values (Δ^17^O represents a deviation from the terrestrial fractionation line, defined by Δ^17^O = δ^17^O − 0.52 × δ^18^O where δ^17^O and δ^18^O denote permil deviations of ^17^O/^16^O and ^18^O/^16^O ratios, respectively, from terrestrial standard values. ε^54^Cr and ε^50^Ti denote parts per 10,000 deviations of ^54^Cr/^52^Cr and ^50^Ti/^48^Ti ratios, respectively, from terrestrial standard values) (Trinquier et al. 2009; Warren 2011; Zhang et al. 2012; Kruijer et al. 2019). These techniques are generally used to identify the meteorite groups, and critically the Martian meteorites. In the case of the captured asteroid scenario where Phobos and Deimos may have distinct isotopic signatures, isotopic measurements can also be used to identify if Deimos materials are present.

### In the case of the captured asteroids scenario

In the case of the captured asteroid scenario, characterization of the returned samples is essential to understand the nature of the volatiles supplied to terrestrial planets in the inner solar system (Fig. 2). Primitive materials like carbonaceous chondrites are easily recognized by textual features, and it is possible to distinguish different carbonaceous chondrite groups based on the bulk chemical compositions of the returned samples. Petrological and isotopic studies on any extant chondrules, refractory inclusions, or matrix will also constrain the asteroidal geological history and classification.

Bulk and microscale δ^15^N and δD (and possibly, δ^13^C) values are useful for understanding the origin of the asteroid/comet because ^15^N and ^2^H (D) enrichments are regarded as the characteristics of their exotic origin from the cold outer solar system (Charnley and Rodgers 2002; Marty 2012; Füri and Marty 2015; Kebukawa et al. 2020). Marty et al. (2016) reported the diagram profiles between the bulk elements (^2^H, ^13^C, ^15^N) and noble gas to discuss the origins of these. In addition, magnetism studies can be used to determine the formation distance from the Sun (Bryson et al. 2020). Abundances of isotopically anomalous grains, so-called presolar grains, such as silicates, oxides, SiC, graphite, and diamond are indicators of the primitiveness as well as the degree of thermal metamorphism/aqueous alteration of chondritic meteorites (e.g., Leitner et al. 2020).

Analyses of organic matter for its chemical and isotopic compositions also provide crucial information about the primordial materials and their evolution in the captured asteroid. For example, compositions of IOM (e.g., C/N and C/H ratios, and δD and δ^15^N values) and compositions of amino acids (e.g., total concentrations and β-alanine/glycine ratios) provide information about the type and thermal history of asteroids (Alexander et al. 2007; Elsila et al. 2016a). Considering a potential hypothesis, it is an interesting quest to understand the history of organic astrochemical processes mediated by a migrated D-type asteroid (Brown et al. 2000; Fujiya et al. 2019). The molecular compositions and isotopic compositions will be useful to constrain the low-temperature chemical reactions in the early solar system and to constrain the aqueous reactions in asteroids, comparing these compositions with those of meteorites. Substantial nano-scale heterogeneities in δD and δ^15^N values in the microorganic matter and lithological variations would be important characteristics indicating primitive materials such as carbonaceous asteroids and comets (Busemann et al. 2006; Alexander et al. 2007; Hashiguchi et al. 2015; Kebukawa et al. 2020).

Chronological information obtained from radiometric dating of the returned samples along with crater counting of Phobos’ surface can shed light on the evolution of the captured asteroid (Schmedemann et al. 2014). The formation ages of primary materials, such as refractory inclusions and chondrules which formed in the solar nebula, can be measured using the ^26^Al–^26^Mg systematics unless it was disturbed by parent body processes like thermal metamorphism and aqueous alteration (e.g., Nakashima et al. 2015; Kawasaki et al. 2019). The timing of thermal and/or shock metamorphism can be constrained from ^39^Ar–^40^Ar (^40^K–^40^Ar) dating (e.g., Mazor et al. 1970; Trieloff et al. 2003; Bogard 2011). The timing of aqueous alteration can be inferred from ^53^Mn–^53^Cr ages of aqueously formed minerals such as carbonates and Fe-rich olivine (Fujiya et al. 2012; Doyle et al. 2015). These radiometric ages could be compared with the surface ages estimated from crater counting on the Phobos surface to constrain when Phobos was captured by Mars’ gravity.

### In the case of the giant impact scenario

In the case of the giant impact scenario, the mineral phases, chemical compositions, and texture of the returned samples can constrain the scale and condition of the giant impact (Fig. 3). The extent of volatile loss can quantitatively be evaluated using stable isotopic tracers for moderately volatile elements (MVE) such as Zn, Ga, and Rb which are known to highly fractionate by volatilization following a giant impact (e.g., Paniello et al. 2012; Kato and Moynier 2017; Pringle and Moynier 2017). Because of the moderate isotopic variations observed among chondrites compared to the fractionation produced during evaporation (e.g. Pringle et al. 2017; Sossi et al. 2018), we expect to find unambiguous evidence for or against the giant impact scenario if the impactor material is chondritic, and the isotopic compositions of the impactor would have little impact on our estimation of the extent of evaporation.

Analyses of organic materials, which would have been derived from meteorites and/or from Mars after the formation of Phobos, will provide information about the materials that delivered water and organic materials to Mars as well as the history of Martian environments. Without the exact sample data from the Phobos surface, we still do not know yet about the fate of “original” organic matter after a hypothetical accretion process on the Martian system. Since the time of the Apollo mission in the 1970s, we have shared the knowledge of Earth’s moon, together with a lot of lessons learned including appropriate sample assessments, analytical procedures, and development of infrastructures through the sample return mission (Taylor 1975; McCubbin et al. 2019). Thus, in the case of the giant impact scenario, we should investigate the possibility of indigenous formation of organic matter as we did for lunar samples (e.g., Hare et al. 1970; Harada et al. 1971; Elsila et al. 2016b) and the compositions will provide complementary information about the formation scenario of Phobos.

The age of the Phobos-forming catastrophic impact event can be obtained from ^238,235^U–^206,207^Pb dating (Terada et al. 2018). We can also obtain a ^87^Rb–^87^Sr model age of the timing of the impact associated with volatile loss and fractionation between Rb and Sr (Amsellem et al. 2020).

### To investigate the surface processes on Phobos

Surface processes such as space weathering and gardening of the Phobos regolith will also be investigated from the returned samples (Fig. 4). Especially, a comparison between samples collected by the C-sampler and P-sampler will be important for understanding the influence of surface processes on this small airless body. Detailed observations of the surfaces of the returned samples can detect morphological and/or mineralogical evidence for space weathering and micrometeorite bombardment (Noguchi et al. 2011; Nakamura et al. 2012; Matsumoto et al. 2015). The space-weathering features on grain surfaces are prone to alteration on Earth even if the samples are kept in a vacuum chamber. Quantification of Fe valence states and the comparison with a time interval of a few month will enable us to evaluate the pristineness of the sample surface.

The cosmic-ray and solar wind exposure ages can be obtained from relative and absolute abundances and isotopic compositions of noble gases (Eberhardt et al. 1970; Wieler et al. 1996; Nagao et al. 2011). These exposure ages provide valuable information about the residence time of small particles and the ages of surface terrains on Phobos. The comparison between the extent of space weathering and exposure ages on one hand, and the surface spectra of the sampling sites on the other hand, can reveal whether the spectral variability on the Phobos surface is a consequence of space weathering, which is considered to result from exposure of cosmic ray and/or solar wind, and micrometeorite bombardment. The ^39^Ar–^40^Ar ages of the returned samples may record the timing of formation of surface terrains, and thus, they can be compared with the surface ages estimated from crater counting (Park et al. 2015; Jourdan et al. 2017).

### To shed light on the surface evolution of Mars

**Fig. 5:**
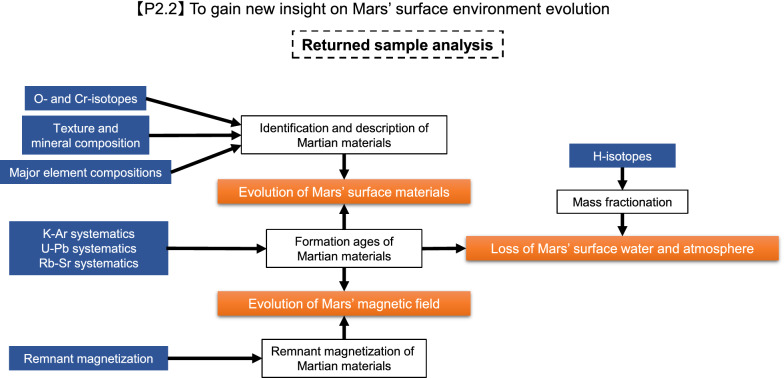
Flowchart of sample analyses together with remote sensing observations to understand the surface evolution of Mars (objective 2.2 in Table 1)

Constraints on the surface evolution of Mars could be placed if Martian materials ejected to Phobos are detected in the returned samples (Fig. 5). Such Martian materials could be found by petrographic and mineralogical observations (e.g., Fe/Mg and Fe/Mn ratios of mafic silicates, and anorthite component; Karner et al. 2003, 2006), and their exact origin could be further corroborated by measuring O, Ca, Ti, and Cr isotopic compositions. Since Martian materials are predicted to have been ejected throughout the entire history of Mars, their formation ages obtained by ^39^Ar–^40^Ar, ^87^Rb–^87^Sr and ^238,235^U–^206,207^Pb dating are essential information about Mars’ surface evolution through time. Along with the radiometric dating, mineralogical/petrological observations of the Martian materials will provide new insights into the compositions of the surface materials and their evolution. Hydrogen isotopic compositions of phosphate and hydrous minerals, both of which contain hydroxyl or H_2_O, can provide detailed knowledge about when and how the surface water and atmosphere of Mars were lost (e.g., Greenwood et al. 2008). In addition, any measurement of remnant magnetization of the Martian materials would be quite intriguing. Because Martian meteorites typically underwent shock metamorphism to > 5 GPa, which would reset any remnant magnetism (Rochette et al. 2001; Bezaeva et al. 2007), the Martian materials ejected to Phobos with lower shock pressures could provide unique clues to better understand the possible evolution of the Martian magnetic field over the planet’s history.

## Sample analyses

We will first observe the returned samples under an optical microscope for initial screening. Selected grains will be analyzed using Raman spectroscopy and Fourier-Transform Infrared Spectroscopy (FT-IR) for further characterization of the samples, and studied for their magnetism using a superconducting quantum interference device (SQUID). Then, we will determine which grains to be processed for inorganic/organic analyses. In the following, the procedures of inorganic and organic analyses are described separately.

The MMX sample allocation committee will make decisions on allocation. Lead scientists (a call for applications will be announced in the future) will decide what kind of measurements should be done.

### Inorganic analyses

**Fig. 6:**
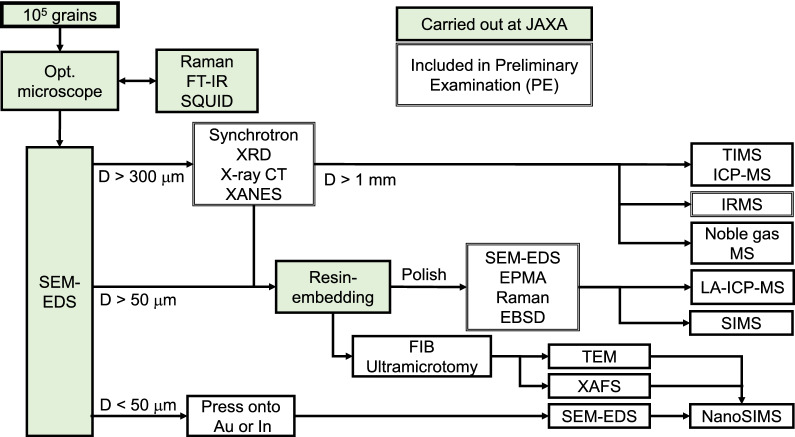
Working flow of inorganic analyses. Analyses that will be included in PE are shown by double-line boxes

The proposed protocols for inorganic analyses are shown in Fig. 6. For grains to be processed for inorganic analyses, we will perform mineralogical observations and qualitative analyses of chemical compositions of the sample grains using a Scanning Electron Microscope (SEM) equipped with an Energy-Dispersive X-ray Spectrometer (EDS) (Nakamura et al. 2011).

#### In-situ analyses

Selected grains larger than 1 mm as well as smaller ones not suitable for “grain-by-grain” bulk analysis will be embedded in the epoxy resin EMBED-812 (or something similar) and polished for in situ analyses using, e.g., Secondary Ion Mass Spectrometry (SIMS) and Laser-Ablation (LA) Inductively Coupled-Plasma Mass Spectrometry (ICP-MS). Before polishing, we will perform X-ray diffraction (XRD) analyses to identify mineral phases in the grains (Nakamura et al. 2011). XRD analyses can be combined with X-ray microtomography observations, and successive 3D-computed tomography (CT) images can construct the internal three-dimensional structures and help us to recognize what minerals are enclosed within the grains (Tsuchiyama et al. 2011). The density and porosity of individual grains can also be determined from the 3D-CT images. When polishing the samples, these 3D-CT images will be utilized to expose particular mineral phases of interest on the polished surface. These techniques have been used for Itokawa samples smaller than 500 μm returned by the Hayabusa spacecraft, and there are lessons learned from the Hayabusa sample analyses. In addition, we will perform X-ray Absorption Near Edge Structure (XANES) analyses for Fe to monitor a possible change in the Fe valence states resulting from terrestrial alteration (Monkawa et al. 2006).

Subsequently the samples will be analyzed using SEM–EDS before further analyses. These initial analyses could also identify phases highly susceptible to alteration, or destruction by epoxy embedding, necessitating alternative analytical protocols. Further characterization of the samples, e.g., quantitative analyses of chemical compositions, observations of crystallography, and identification of mineral phases, will be performed using an Electron Probe Micro-Analyzer (EPMA), Electron Back Scatter Diffraction (EBSD), and Raman spectroscopy.

The SIMS and LA-ICP-MS techniques will be utilized to obtain chemical and isotopic compositions in small regions of the returned samples with a spatial resolution of a few to tens of micrometers. Concentrations of trace elements such as rare earth elements (REEs) in mineral phases can be measured by the LA-ICP-MS technique (e.g., Joy et al. 2006; Yokoyama et al. 2011; Stead et al. 2017). Oxygen-isotope measurements by SIMS have widely been applied to individual mineral grains in a polished thin section. Since an analytical precision of ~ 0.3‰ on Δ^17^O values have been achieved for the Itokawa samples as small as tens of micrometers, we could distinguish the Δ^17^O difference of ~ 0.3‰ between terrestrial and Martian samples by repeated analyses (e.g., Yurimoto et al. 2011; Nakashima et al. 2013). In addition, the SIMS and (LA)-ICP-MS methods enable radiometric dating of individual minerals. For instance, short-lived radionuclide chronometry such as ^26^Al–^26^Mg, ^53^Mn–^53^Cr, and ^182^Hf–^182^W systematics has been utilized for dating of refractory inclusions and chondrules (e.g., Young et al. 2005; Nakashima et al. 2015; Kawasaki et al. 2019), carbonate and Fe-rich olivine (e.g., Fujiya et al. 2012; Doyle et al. 2015), and zircon grains (Srinivasan et al. 2007; Koike et al. 2017), respectively. We can also analyze ^238,235^U–^206,207^Pb systematics on U-bearing mineral grains such as zircon and phosphate using SIMS and LA-ICP-MS methods (e.g., Chang et al. 2006; Terada et al. 2018).

Ultrathin sections of the returned samples embedded in resin or sulfur will be produced by ultra-microtomy or Focused Ion Beam (FIB) techniques. The embedding material will be tailored to the proposed analyses. Epoxy will be used for inorganic analyses. These ultrathin sections will be observed for space-weathering rims on the sample’s surface using a Transmission Electron Microscope (TEM) (Noguchi et al. 2011). Space-weathering rims on the Itokawa samples as small as tens of micrometers have been observed in a similar manner, and the analyzed grains were handled without reacting with atmospheric oxygen and water vapor during embedding, ultra-microtomy, and transportation to the instrument. In the case of the captured asteroid scenario, the fine-grained matrix of the samples will also be observed using a TEM (Noguchi et al. 2017). In addition, we will analyze these ultrathin sections for chemical speciation of, e.g., multivalent elements such as Ti, V, Cr, and Fe to investigate their valent states by X-ray Absorption Fine Structure (XAFS) (Sutton et al. 2020). The same samples from which the ultrathin sections are extracted using FIB will be analyzed using a high spatial resolution SIMS (NanoSIMS) for further information about their isotopic compositions.

For grains smaller than ~ 50 μm, the samples may be prepared by pressing them onto ultrapure Au or In foil for further analyses to search for presolar grains (Noguchi et al. 2017). Presolar O-rich minerals (i.e., silicate and oxide) and SiC can be detected by ion imaging of O and C isotopic compositions, respectively, acquired using a NanoSIMS (Hoppe et al. 2017).

#### Bulk analyses of chemical compositions and noble gas for large (~ 1 mm) grains

Grains larger than ~ 1 mm can be analyzed for bulk chemical compositions or stable isotopic compositions of, e.g., O, Ti, Cr, and Zn. Before bulk analyses, we must check for any apparent contamination from the laboratory or spacecraft using SEM. Grains larger than a few hundreds of microns may contain a variety of minerals within them. For bulk Ti-, Cr-, and Zn-isotope analyses, it is important to recognize mineral phases and their Ti, Cr, and Zn abundances in the grains to ensure that the required analytical precisions can be achieved. Thus, we will perform XRD analyses to identify mineral phases before bulk analyses.

The bulk chemical compositions will be measured by ICP-MS and may possibly already discriminate between a captured asteroid (possibly chondritic) and Mars/impactor-derived materials (non-chondritic, fractionated). In the captured asteroid scenario, a combination of volatile, moderately volatile, and refractory element contents is useful to distinguish different carbonaceous chondrite groups (Friedrich et al. 2002). In the giant impact scenario, measurements of highly siderophile element (HSE) concentrations would be interesting. Since the abundances of HSEs in the terrestrial and Martian mantles are similar and about 200 times lower than the chondritic abundances, the proportion of the impactor (chondritic) materials consisting of Phobos might be evaluated from HSE concentrations (Walker 2009). In addition, MVE would be expected to be highly depleted as observed for the Moon compared to any groups of chondrites or Martian meteorites (shergottites, nakhlites, and chassignites: SNC meteorites) (Day and Moynier 2014).

A noble gas MS can be used to investigate cosmogenic nuclides and solar wind components which can shed light on surface processing on Phobos. Helium, Ne, and Ar isotopic compositions have been obtained for the samples as small as tens of micrometers (corresponding to ~ 0.1 μg) in size from asteroid Itokawa, and the abundances of solar wind and cosmogenic components were determined from the Ne isotopic ratios (Nagao et al. 2011). There are lessons learned from the Hayabusa sample analyses where the samples were handled in a clean chamber at the curation facility of JAXA. The chamber was filled with pure N_2_ gas and concentrations of noble gases were 2–5 orders of magnitude lower than those in the atmosphere. On the other hand, Kr- and Xe-isotope analyses require higher sample amounts. Assuming chondritic Kr and Xe concentrations of ~ 10^–9^ cm^3^STP g^−1^ (Busemann et al. 2000) and the detection limit on ^132^Xe of 10^–15^ to 10^–16^ cm^3^STP (Crowther and Gilmour 2012; Meshik et al. 2014), sample amounts of ~ 1 mg, comparable to the sample amounts for O-, Ti-, and Cr-isotope analyses, would be required.

#### Bulk isotope analyses

The most powerful tool to investigate the origin of Phobos’ building blocks would be stable isotopic ratios of elements like O, Ca, Ti, and Cr. Although bulk isotope analyses of these elements have not been tried for the Itokawa samples due to their small sample amounts, such analyses may be possible for samples returned by Hayabusa 2 and OSIRIS-REx missions from C-type asteroids. We can expect to learn lessons from these missions, as such whether these measurements can be done for highly brecciated samples.

Δ^17^O values of a few mg silicate samples (approximately 1 mm^3^) can be measured by a laser fluorination system with an analytical precision of less than 0.01‰, enough to distinguish between different meteorite groups (e.g., Herwartz et al. 2014; Young et al. 2016; Greenwood et al. 2018). The Cr isotopic composition of extraterrestrial materials is usually measured by ICP-MS or Thermal Ionization Mass Spectrometry (TIMS) (e.g., Mougel et al. 2018; Zhu et al. 2020), while Ti isotopic composition is measured by ICP-MS (e.g., Trinquier et al. 2009). The mass of Cr and Ti required for such measurements at high precision is roughly around 1 μg, which represent a few mg of samples whichever it is chondritic (e.g., ~ 3000 ppm Cr and 500 ppm Ti) or differentiated like shergottites (~ 700 ppm Cr and 6000 ppm Ti) (Brown et al. 2000; Taylor et al. 2002; Hibiya et al. 2019). Thus, a combined Cr and Ti analysis for the returned samples larger than 1 mm seems feasible.

An isotopic dichotomy between CCs and NCs is also clearly demonstrated by Mo isotopic ratios in ε^95^Mo versus ε^94^Mo space (ε^95^Mo and ε^94^Mo denote parts per 10,000 deviations of ^94^Mo/^96^Mo and ^95^Mo/^96^Mo, respectively, from terrestrial standard values), where discrete regression lines can be drawn for CC and NC data (Kruijer et al. 2017; Budde et al. 2019). In such a diagram, Mo isotopic ratios produced by mixing two Mo reservoirs plot on a straight line connecting the two Mo endmembers (Budde et al. 2019). This is a major advantage for using Mo isotopic ratios to investigate the origin of the returned samples, because they might have such intermediate compositions in the case of the giant impact origin where the composition of the Phobos building blocks is determined by mixing between the Martian and impactor materials. However, a sample amount of ~ 500 mg with 1 ppm Mo, typical for carbonaceous chondrites (Burkhardt et al. 2014), is required at present to obtain a precision of ~ 0.1*ε* using ICP-MS, sufficient to distinguish between CCs and NCs. Martian meteorites may have even lower Mo contents (Taylor et al. 2002). Therefore, Mo isotopic analyses of the returned samples might be unrealistic.

Stable isotopic composition of MVE is usually measured by ICP-MS. For the stable isotopic composition of MVE such as Zn, the amount of sample required would vary considerably depending on whether it is chondritic (> 100 ppm) or volatile depleted like lunar basalts (~ 1 ppm) (Paniello et al. 2012). The most recent analytical development requires ~ 10 ng of Zn to produce high precision Zn isotopic data and ~ 5 ng for slightly lower precision (van Kooten and Moynier 2019). This, therefore, represents less than a mg for a chondritic composition and ~ 10 mg for a volatile depleted composition. For other MVE, such as Ga and Rb, isotopic measurements presently require ~ 100 ng for high precision which represents 50–100 mg of volatile depleted material (and a few mg for chondritic composition) (e.g., Kato et al. 2017; Pringle and Moynier 2017; Moynier et al. 2021). However, while the isotopic measurement of Zn has been actively optimized to utilize a minimum amount of material, those of other MVE have not, leaving the opportunity for improvements in the future and possibly in time for analysis of MMX material.

If the returned samples are chondritic materials of a captured asteroid origin, bulk N and C isotopic compositions will be measured for a few mg grains using an isotope ratio mass spectrometer (IRMS) (Grady et al. 2002). A stepped-combustion system enables us to attribute the N and C abundances and isotopic compositions obtained during different temperature intervals to discrete components such as organic species, carbonates, and presolar grains (e.g., SiC, graphite, and nanodiamond).

#### Radiometric dating for bulk samples

Radiometric dating for bulk samples with ICP-MS or TIMS can also be applied. ^53^Mn–^53^Cr, ^87^Rb–^87^Sr, and ^238,235^U–^206,207^Pb systematics will provide model ages of the samples by assuming their initial isotopic compositions at the time of isotopic closure (e.g., Lugmair and Shukolyukov 1998; Bouvier et al. 2018; Amsellem et al. 2020). In the captured asteroid scenario, for example, the bulk ^53^Cr/^52^Cr and ^55^Mn/^52^Cr ratios of the returned samples may plot on a regression line defined by bulk chondrite data, which has been thought to represent the timing of Mn/Cr fractionation in the protoplanetary disk (Trinquier et al. 2008). In the giant impact scenario, ^87^Rb–^87^Sr and ^238,235^U–^206,207^Pb systematics can date the timing of the giant impact. We will possibly combine multiple grains to obtain their ages assuming that they have similar ages.

A noble gas MS can be utilized to obtain ^39^Ar–^40^Ar ages of the returned samples. Previously, three Itokawa grains with a total mass of 2 μg were analyzed as a group for Ar isotopic compositions, which gave a ^39^Ar–^40^Ar age of 1.3 ± 0.3 Ga (Park et al. 2015). Another single Itokawa grain, which undergone 15–25 GPa impact shock pressure, has a ^39^Ar–^40^Ar age of 2.3 ± 0.1 Ga (Jourdan et al. 2017). However, required sample amounts and corresponding errors are highly dependent on the K contents of the samples. ^39^Ar–^40^Ar (K–Ar) ages of carbonaceous chondrites are sparse and difficult to measure due to the presence of the “phase Q” noble gases (Busemann et al. 2000), however, the ages of the samples returned by the Hayabusa 2 spacecraft from the C-type asteroid Ryugu may be measured. Thus, we expect to learn from the Hayabusa 2 sample analyses about sample mass required and the influence of the phase Q gases.

### Organic analyses

#### Potential features of organic matter in Phobos samples

Potentially, contents of soluble organic matter such as amino acids and carboxylic acids through target analysis are useful indicators to evaluate the extent of the thermal/aqueous processes in the asteroid (e.g., Glavin et al. 2011, 2018). Non-target high-resolution mass spectrometry of soluble organic compounds will be useful to evaluate the organic synthesis reactions in the asteroid or before the accretion of the asteroid (Schmitt-Kopplin et al. 2010; Naraoka et al. 2017; Naraoka and Hashiguchi 2019; Orthous-Daunay et al. 2019). Micro-scale analysis, e.g., Time of Flight (ToF)-SIMS and Desorption Electrospray Ionization (DESI)-MS of the distribution of organic compounds in the sample will be more useful for the evaluation of the synthesis and evolution of asteroid organic matter (e.g., Naraoka et al. 2015; Naraoka and Hashiguchi 2018). The analyses of labile organic compounds are substantially important in the case of the captured asteroid origin since they have the potential to be the first pristine organic matter from the outer solar system. The candidate list of key organic analyses through the MMX mission was preliminarily shown by Usui et al. (2020).

#### Non-destructive and semi-destructive analysis

Microscopic analyses and chemical extraction analyses are two important approaches to characterize organic matter in Mars moon samples. The comprehensive microscopic analyses include X-ray CT (e.g., Tsuchiyama et al. 2011; Friedrich et al. 2016, 2019), SEM–EDS, TEM-EDS (e.g., Uesugi et al. 2014, 2019), micro-Raman (e.g., Busemann et al. 2007; Kitajima et al. 2015), NanoSIMS (e.g., Hoppe 2006; Ito et al. 2014; Pant et al. 2018), and XANES (e.g., Orthous-Daunay et al. 2010; Derenne and Robert 2010; Yabuta et al. 2014; Kebukawa et al. 2019). These analyses will use grains as small as 1 mm to acquire basic and precise information of the organic matter. The SEM–EDS and TEM-EDS are for the basic characterization of the distribution and local compositions of carbonaceous matter. If micro-scale organic aggregates are included, a part of them will be analyzed by micro-Raman spectroscopy to understand the maturation and graphitization of the carbonaceous matter. Other parts of the organic-rich area will be analyzed by NanoSIMS for understanding the local enrichments in ^2^H (D), ^13^C, and ^15^N in different types of organic matter. Most of these analyses will use thin sections of small samples and thus could be conducted along with some inorganic analyses or using the same sample as for some inorganic analyses (see “In-situ analyses” in “Inorganic analyses” section). For organic analyses, we prefer to use sulfur as the embedding material to avoid organic contamination. The distributions of minerals in these samples will also be investigated with SEM, TEM, and SIMS to understand the spatial relations of minerals and organic compounds.

#### Volatile and semi-volatile gas analysis

There are scientific heritages acquired through the previous sample return mission in terms of “initial” volatile and semi-volatile organic gas recovery generated from an asteroid returned sample (e.g., Okazaki et al. 2017; Sawada et al. 2017). After the online purification of target organic molecules in the vapor phase, volatile and semi-volatile organic gases are processed to non-destructive and destructive analytical procedures without exposure to terrestrial ambient air. The former representative example is a method of Cavity Ring-Down laser absorption Spectroscopy (CRDS) (e.g., Scherer et al. 1997; Berden et al. 2000; Tittel et al. 2003). The latter example is a gas chromatography/mass spectrometry (GC/MS) equipped with appropriate interfaces of head-space sampler (HS), solid-phase microextraction (SPME), and thermal desorption (TD) (e.g., Huang et al. 2007; Aponte et al. 2015; Takano et al. 2020). Low molecular weight hydrophilic and hydrophobic molecules (e.g., organic acids, amines, and hydrocarbons) can be introduced in the GC with derivatization (e.g., Aponte et al. 2016) or without derivatization process (e.g., Snyder et al. 2011). The volatiles would be released by gentle heating of the moon sample in a vessel.

#### Extractable organic analyses and the importance of molecular separation

**Fig. 7:**
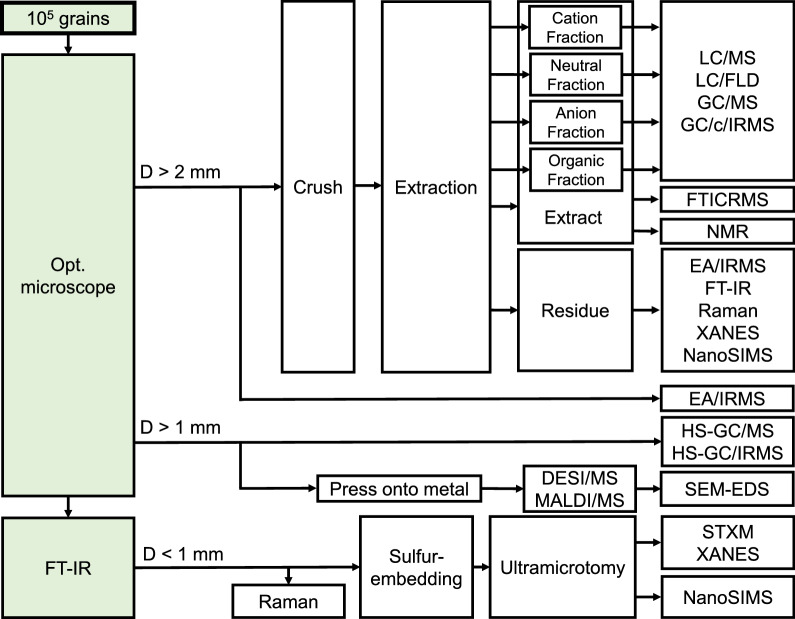
Working flow of organic analyses

A huge variety of organic compounds has been detected in carbonaceous chondrites (i.e., more than 50,000 compositions) (Schmitt-Kopplin et al. 2010). Thus, the identification of organic compounds is not easy but substantially important. Figure 7 represents an entire framework and conceptual design of the non-target and target analyses on the basis of hydrophilicity and hydrophobicity of organic molecules in each procedure. Some of the procedures are closely related to mass spectrometry as destructive online methods, coupling with semi-destructive methods of spatial high-resolution imaging mass spectrometry (e.g., Takáts et al. 2004; Cornett et al. 2007; McDonnell and Heeren 2007; Watrous and Dorrestein 2011).

“Chromatography” is one of the most fundamental and useful analytical tools for comprehensive molecular separation and isolation, in particular for the separation between isomers, even in gas-phase and liquid-phase extracts (e.g., Snyder et al. 2011; McNair et al. 2019). Practically, specific contents of soluble organic compounds (e.g., amino acids, amines, nucleobases, and hydrocarbons) in the extracts will be primarily separated by gas chromatography and liquid chromatography (LC) and/or multi-dimensional separation system (i.e., 2D-GC, 2D-LC), resulting in accurate molecular identification and assignment as seen in the abovementioned pioneering works. A combination of LC with GC will give us robust baseline resolution for target molecules (e.g., Takano et al. 2015). If we confirm the chromatographic baseline resolution of target molecules and high amounts of specifically interested extractable organic compounds (e.g., amino acids and sugars), their compound-specific C and N isotope compositions will be analyzed with gas chromatography/combustion/isotope ratio mass spectrometry (GC/C/IRMS) (e.g., Aponte et al. 2014; Elsila et al. 2016b; Chan et al. 2016; Glavin et al. 2018; Furukawa et al. 2019).

To perform a molecular identification, the mass spectrometric assignment is required in both non-target and target analysis via various ion sources and detectors (e.g., Gross 2006). In the target-specific analysis such as the analysis of specific amino acids, amines, and sugars, chromatographic separation is coupled with several types of mass spectrometry including high-resolution mass spectrometry and mass spectrometry associated with molecular fragmentation for exact molecular identification (Pizzarello et al. 2001; Glavin et al. 2010, 2011; Aponte et al. 2014, 2015, 2016; Naraoka et al. 2017; Furukawa et al. 2019). Approximately 1–10 mg of samples are needed for the analysis of such extractable organic compounds since the contents of these organic compounds are not expected to be high. Alternatively, a liquid chromatography/fluorescence detection and mass spectrometry (LC-FLD/MS) system is also promising for the evaluation of derivatized organic molecules (e.g., Glavin et al. 2010; Hamase et al. 2014; Furusho et al. 2020). Naraoka et al. (2012) reported the capability of ultra-small-scale analysis (~ femto mol scale) for extraterrestrial chiral amino acids and the evaluation of the Hayabusa sample from Itokawa.

Mass spectrometry coupled with semi-destructive methods of spatial high-resolution imaging is a rather new and useful analysis for extraterrestrial samples (e.g., Takáts et al. 2004; Cornett et al. 2007; McDonnell and Heeren 2007; Watrous and Dorrestein 2011). This will be performed directly to 0.1–1 mg grains to understand the distribution of organic compounds in the sample using DESI and matrix-assisted laser desorption/ionization (MALDI) combined with high-resolution mass spectrometry (Naraoka et al. 2019).

For non-target analysis, water extracts of the samples will further be analyzed using a Fourier-transform ion cyclotron resonance mass spectrometry (FTICR-MS) for high mass-resolution analysis of organic compounds (Hertzog et al. 2019). The other inspection using high mass resolution Orbitrap mass spectrometry would support further organic profiles and new findings (e.g., Smith et al. 2014; Oba et al. 2019, 2020; Orthous-Daunay et al. 2020). Extraction residues of these organic compounds will be demineralized and analyzed with ultra-small-scale elemental analysis/isotope ratio mass spectrometry (nano-EA/IRMS; Ogawa et al. 2010, 2020) for elemental compositions and isotope ratios of, e.g., C, N, and S. In addition, these residues will be analyzed by FT-IR, Raman spectroscopy, XANES, EA/IRMS, and NanoSIMS to obtain information about spatial distribution of chemical species and their isotopic compositions (Kebukawa et al. 2020). Inorganic salt profiles will be clarified by small-scale ion chromatography (Yoshimura et al. 2020). In combination with mass spectrometry, small extractable organic compounds will be extracted using multiple solvents (i.e., aqueous and organic solvents) after crashing. The aqueous extract will first be analyzed with high field nuclear magnetic resonance (NMR) for C and H chemical shifts of polar extractable organic compounds (Hertkorn et al. 2015).

## Possible Martian materials

**Fig. 8:**
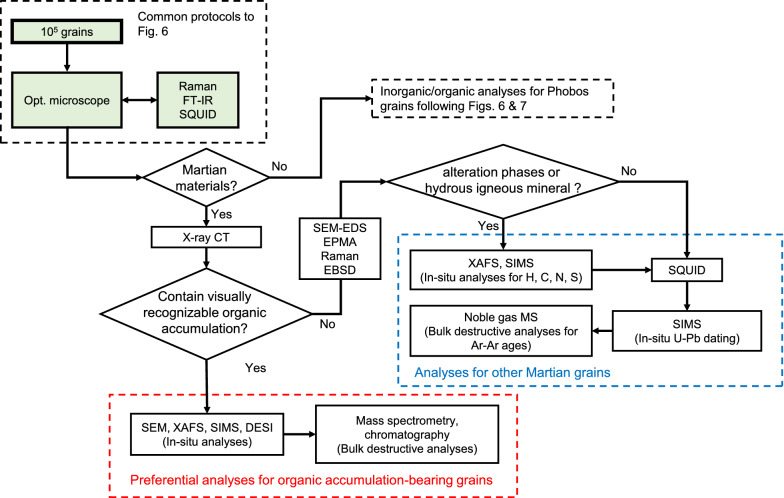
Working flow of Martian material analyses

As mentioned previously, we may find Martian materials ejected by asteroidal impacts from Mars in the returned Phobos regolith. These Martian materials may include not only igneous phases but also alteration materials that formed under the presence of water such as clay minerals, carbonates, sulfates, and chlorides as found on the Martian surface (Ehlmann et al. 2011). Such Martian materials will be processed separately (Fig. 8). The Martian igneous materials likely show similar chemical and mineralogical trends to those of Martian meteorites. Thus, their characteristic chemical compositions, such as Mn/Fe ratios, will be useful to identify the Martian igneous materials (Papike et al. 2009). Even if Phobos is formed by a giant impact, Martian igneous materials such as olivine and pyroxene could be distinguished from the Phobos’ endogenous materials because the chemical compositions of the Phobos’ endogenous materials are likely a mixture of Martian and impactor materials and are distinct from Martian igneous materials. On the other hand, we can identify the Martian alteration materials by comparing their morphology, mineralogy, and chemistry to alteration materials found in the Martian meteorites, such as carbonate globules in Allan Hills (ALH) 84,001 and carbonate-clay-bearing veins in nakhlites (Bridges et al. 2019). Note that, however, these minerals are common in carbonaceous chondrites and in particular the Tagish Lake meteorite (Zolensky et al. 2002; Brearley 2006), and thus, it may be difficult to identify these minerals derived from Mars in the case of the captured asteroid scenario. Hence, whether the both types of Martian materials can be identified using non-destructive techniques like an optical microscope, FT-IR, SEM–EDS, EPMA, and micro-Raman spectroscopy during curation depends on the origin of Phobos. Their Martian origin could be unambiguously confirmed by O-isotope analysis.

Among the expected mineral types of Martian grains, carbonates, sulfates, and clay minerals are important targets to understand the Martian surface environments, because they would preserve ancient records of aqueous environments and provide crucial information about the past geochemical (and possibly, past biochemical) processes on Mars. For example, combined analyses of radiometric ages and chemical and isotopic compositions of volatile elements will reveal temperatures, the extent of volatile loss, abiotic (or potentially biotic) organic synthesis, and redox states with time. Furthermore, the search for potential signatures of past Martian biota is fascinating. The 4 Ga carbonates in ALH 84,001 may have preserved ancient Martian organic materials (e.g., Koike et al. 2020). The discovery of the present living Martian organism in the returned samples is unlikely, because they were sterilized during the impact delivery from Mars to Phobos and subsequent solar and cosmic-ray irradiation to the Phobos surface (Fujita et al. 2019; Kurosawa et al. 2019). However, the shock pressure that the Martian materials on Phobos may have undergone, far lower than that for Martian meteorites (> 5 GPa) (Kurosawa et al. 2018; Hyodo et al. 2019), would not decompose labile organic compounds such as amino acids (Sugahara and Mimura 2014). Therefore, the trace of Martian biomolecules might be preserved on Phobos. Detailed analyses of specific elements (e.g., P and S) and isotopic ratios (e.g., ^13^C/^12^C and ^15^N/^14^N ratios) may provide important data to investigate potential biosignatures (e.g., Delarue et al. 2020). It should be noted that there is a survival bias of organic molecules by the impact. For example, long-chain aliphatic hydrocarbons are easily decomposed than aromatic ones (Montgomery et al. 2016). Even the isotopic signatures (^13^C/^12^C and D/H ratios) of organic materials are likely affected by impact (Mimura et al. 2007).

If we find possible Martian materials in the returned Phobos samples, the first investigation will be to search for the accumulation of organic materials under an optical microscope. If such accumulations are not found under the optical microscope, then X-ray 3D-CT will be utilized to search them on a smaller scale. If such accumulations are discovered, further in situ analyses using an SEM, XAFS, NanoSIMS, and DESI-MS will be performed to characterize them and to investigate the organic synthesis. In addition, if the Martian materials have sufficient amounts of organic materials, mass spectrometry coupled with a chromatographic technique might be applicable to characterize undescribed Martian organic molecules.

The Martian materials without any recognizable organic materials observed by X-ray 3D-CT will be processed for inorganic chemical and isotopic analyses. Alteration materials (e.g., phyllosilicates and carbonates) and hydrous igneous minerals (e.g., apatite) recorded the conditions of the Martian atmosphere and hydrosphere at various timings. After the careful observation using an SEM, EPMA, Raman spectroscopy, and EBSD to select target minerals, in situ XAFS and SIMS analyses will be conducted for volatile elements (e.g., H, C, N, O, and S). In addition, the remnant magnetization of the Martian materials will be measured using SQUID. SQUID analyses should be performed before extensive handling possibly disturbs a weak magnetic field. If these grains also contain U-bearing minerals (e.g., apatite, zircon, and baddeleyite), ^238,235^U–^206,207^Pb dating using SIMS will provide chronological information of the volatile and/or magnetic records. Other radiometric dating, e.g., ^39^Ar–^40^Ar and ^87^Rb–^87^Sr dating could also be applicable for bulk samples if the grains are large enough.

## Preliminary Examination of the returned samples

The nature of the samples returned by MMX (i.e., primitive chondritic or igneous non-chondritic materials), which is linked to the origin of Phobos, is uncertain. This is a significant difference between MMX and Hayabusa 2 samples, because the latter were collected from a C-type asteroid Ryugu, and hence, they are expected to be chondritic materials, although there will be new and important findings on these samples (Tachibana et al. 2014). The feasibilities of analyses, such as bulk isotope measurement and organic analyses, depend on the nature of the samples. Therefore, it is highly desirable that the nature of the returned samples, and possibly, the origin of Phobos are revealed before the samples are processed for detailed analyses in laboratories.

For this reason, we plan to initiate initial analyses (hereafter referred to as Preliminary Examination: PE to avoid confusion) together with curation procedures. The curation and PE teams will be working simultaneously together not only for the science but also for making a catalog. Analyses that will be performed during PE are shown by double-line boxes in Fig. 6. The purposes of PE are to identify basic aspects of the collection, to characterize the returned samples to guide later analyses, and to give feedback to the curation procedures. The relatively large mass of the returned samples (> 10 g) enables us to prepare an aliquot for PE, and the amount of the aliquot for PE will be roughly 1% of the total mass of the returned samples. If there are variations in their visual aspects, such as colors and morphology, we will select as diverse grains as possible. For the selected grains, synchrotron XRD and Fe-XANES analyses will first be performed to identify mineral phases in the grains and to evaluate the influence of terrestrial alteration. Depending on the grain sizes, these samples will be further analyzed either for oxygen isotopes using IRMS or for chemical compositions using EPMA. The combination of O isotopic characteristics, chemical compositions, and mineral phases seems capable of determining at least whether the samples are primitive chondritic or igneous non-chondritic materials.

## Conclusions

Martian Moons eXploration (MMX) is a sample return mission from a Martian satellite Phobos. We, the Sample Analysis Working Team (SAWT) members, are now designing the initial analysis plan of the returned samples to achieve the scientific objectives of MMX and to maximize its scientific results. It is challenging but exciting to design the analysis plan without any definitive information about what kind of materials will be coming back to Earth, because the origin of the Martian moons is unknown. However, SAWT is working together with the remote sensing observation teams of MMX and will try to acquire crucial information about the materials on Phobos and clues for its origin before the samples are returned to Earth. Not only in situ analyses but also bulk analyses, which typically require more than a few mg of samples, can be performed taking advantage of the expected relatively large mass (> 10 g) of the returned samples. We will separate a few aliquots from the whole samples for inorganic and organic analyses as well as for Preliminary Examination (PE) which will be performed together with curation and give feedback to the curation procedures. We are also considering how to identify possible Martian materials from the dominant Phobos endogenous materials by PE. Between now and receiving Phobos samples in 2029 we expect that new generation Mars rovers will have collected more information about the nature of Martian sediments, we anticipate improvements in analytical capabilities in elemental and isotopic analysis that will optimize our PE, and we will have learned lessons from the curation and examination of Hayabusa 2 and OSIRIS-REx materials. We believe that the sample analyses will unravel not only the origin of the Martian moons but also the evolution of the Mars–moon system, and that our knowledge to be obtained from MMX will guide future sample return missions from planetary bodies, especially from Mars.

## Data Availability

Not applicable.

## Abbreviations

JAXA: Japan Aerospace Exploration Agency
MMX: Martian Moons eXploration
SAWT: Sample Analysis Working Team
ICSU: International Council for Scientific Unions
COSPAR: Committee of Space Research
WIS: Wisconsin Range
COSIMA: Cometary Secondary Ion Mass Analyzer
SOM: Soluble organic matter
IOM: Solvent-insoluble organic matter
MEGANE: Mars–moon Exploration with Gamma rays and Neutrons
CC: Carbonaceous chondrite
NC: Non-carbonaceous chondrite
MVE: Moderately volatile elements
SEM: Scanning Electron Microscope
EDS: Energy-Dispersive X-ray Spectrometer
FT-IR: Fourier-Transform Infrared Spectroscopy
SQUID: Superconducting quantum interference device
XANES: X-ray Absorption Near Edge Structure
XRD: X-ray diffraction
ICP-MS: Inductively Coupled-Plasma Mass Spectrometry
HSE: Highly siderophile element
SNC: Shergottites, Nakhlites, Chassignites
TIMS: Thermal Ionization Mass Spectrometry
IRMS: Isotope ratio mass spectrometer
LA: Laser Ablation
CT: Computed tomography
EPMA: Electron Probe Micro-Analyzer
EBSD: Electron Back Scatter Diffraction
REE: Rare earth element
FIB: Focused Ion Beam
TEM: Transmission Electron Microscope
XAFS: X-ray Absorption Fine Structure
ToF: Time of Flight
DESI: Desorption Electrospray Ionization
CRDS: Cavity Ring-Down laser absorption Spectroscopy
GC/MS: Gas chromatography/mass spectrometry
HS: Head-space sampler
SPME: Solid-phase microextraction
TD: Thermal desorption
LC: Liquid chromatography
GC/C/IRMS: Gas chromatography/combustion/isotope ratio mass spectrometry
LC-FLD/MS: Liquid chromatography/fluorescence detection and mass spectrometry
MALDI: Matrix-assisted laser desorption/ionization
FTICR-MS: Fourier-transform ion cyclotron resonance mass spectrometry
EA/IRMS: Elemental analysis/isotope ratio mass spectrometry
NMR: Nuclear magnetic resonance
ALH: Allan Hills
PE: Preliminary Examination

## References

[CR1] Albarède F (2009). Volatile accretion history of the terrestrial planets and dynamic implications. Nature.

[CR2] Alexander CMO’D, Fogel M, Yabuta H, Cody G (2007). The origin and evolution of chondrites recorded in the elemental and isotopic compositions of their macromolecular organic matter. Geochim Cosmochim Acta.

[CR3] Amsellem E, Moynier F, Mahan B, Beck P (2020). Timing of thermal metamorphism in CM chondrites: implications for Ryugu and Bennu future sample return. Icarus.

[CR4] Aponte JC, Dworkin JP, Elsila JE (2014). Assessing the origins of aliphatic amines in the Murchison meteorite from their compound-specific carbon isotopic ratios and enantiomeric composition. Geochim Cosmochim Acta.

[CR5] Aponte JC, Dworkin JP, Elsila JE (2015). Indigenous aliphatic amines in the aqueously altered Orgueil meteorite. Meteorit Planet Sci.

[CR6] Aponte JC, McLain HL, Dworkin JP, Elsila JE (2016). Aliphatic amines in Antarctic CR2, CM2, and CM1/2 carbonaceous chondrites. Geochim Cosmochim Acta.

[CR7] Bardyn A, Baklouti D, Cottin H, Fray N, Briois C, Paquette J, Stenzel O, Engrand C, Fischer H, Hornung K, Isnard R, Langevin Y, Lehto H, Le Roy L, Ligier N, Merouane S, Modica P, Orthous-Daunay F-R, Rynö J, Schulz R, Silén J, Thirkell L, Varmuza K, Zaprudin B, Kissel J, Hilchenbach M (2017). Carbon-rich dust in comet 67P/Churyumov-Gerasimenko measured by COSIMA/Rosetta. Mon Not R Astron Soc.

[CR8] Berden G, Peeters R, Meijer G (2000). Cavity ring-down spectroscopy: experimental schemes and applications. Int Rev Phys Chem.

[CR9] Bezaeva NS, Rochette P, Gattacceca J, Sadykov RA, Trukhin VI (2007). Pressure demagnetization of the Martian crust: ground truth from SNC meteorites. Geophys Res Lett.

[CR10] Bogard DD (2011). K–Ar ages of meteorites: clues to parent-body thermal histories. Geochemistry.

[CR11] Bouvier LC, Costa MM, Connelly JN, Jensen NK, Wielandt D, Storey M, Nemchin AA, Whitehouse MJ, Snape JF, Bellucci JJ, Moynier F, Agranier A, Gueguen B, Schönbächler M, Bizzarro M (2018). Evidence for extremely rapid magma ocean crystallization and crust formation on Mars. Nature.

[CR12] Brearley AJ, Lauretta DS, McSween HY (2006). The action of water. Meteorites and the early solar system II.

[CR13] Brearley A, Jones R, Papike JJ (1998). Chondritic meteorites. Planetary materials. Reviews in mineralogy and geochemistry.

[CR14] Bridges JC, Hicks LJ, Treiman AH, Filiberto J, Schwenzer SP (2019). Carbonates on Mars. Volatiles in the Martian Crust.

[CR15] Brown PG, Hildebrand AR, Zolensky ME, Grady M, Clayton RN, Mayeda TK, Tagliaferri E, Spalding R, MacRae ND, Hoffman EL, Mittlefehldt DW, Wacker JF, Bird JA, Campbell MD, Carpenter R, Gingerich H, Glatiotis M, Greiner E, Mazur MJ, McCausland PJA, Plotkin H, Mazur TR (2000). The fall, recovery, orbit, and composition of the Tagish Lake meteorite: a new type of carbonaceous chondrite. Science.

[CR16] Bryson JFJ, Weiss BP, Biersteker JB, King AJ, Russell SS (2020). Constrains on the distances and timescales of solid migration in the early solar system from meteorite magnetism. Astrophys J.

[CR17] Budde G, Burkhardt C, Kleine T (2019). Molybdenum isotopic evidence for the late accretion of outer solar system material to Earth. Nat Astron.

[CR18] Bunch TE, Chang S (1980). Carbonaceous chondrites-II. Carbonaceous chondrite phyllosilicates and light element geochemistry as indicators of parent body processes and surface conditions. Geochim Cosmochim Acta.

[CR19] Burkhardt C, Hin RC, Kleine T, Bourdon B (2014). Evidence for Mo isotope fractionation in the solar nebula and during planetary differentiation. Earth Planet Sci Lett.

[CR20] Burns JA, Kieffer HH, Jakosky BM, Snyder CW, Matthews MS (1992). Contradictory clues as to the origin of the Martian moons. Mars.

[CR21] Busemann H, Baur H, Wieler R (2000). Primordial noble gases in “phase Q” in carbonaceous and ordinary chondrites studied by closed-system stepped etching. Meteorit Planet Sci.

[CR22] Busemann H, Young AF, Alexander CMO’D, Hoppe P, Mukhopadhyay S, Nittler LR (2006). Interstellar chemistry recorded in organic matter from primitive meteorites. Science.

[CR23] Busemann H, Alexander CMO’D, Nittler LR (2007). Characterization of insoluble organic matter in primitive meteorites by microRaman spectroscopy. Meteorit Planet Sci.

[CR24] Canup R, Salmon J (2018). Origin of Phobos and Deimos by the impact of a Vesta-to-Ceres sized body with Mars. Sci Adv.

[CR25] Carr MH, Head JW (2010). Geologic history of Mars. Earth Planet Sci Lett.

[CR26] Chan HS, Chikaraishi Y, Takano Y, Ogawa NO, Ohkouchi N (2016). Amino acid compositions in heated carbonaceous chondrites and their compound-specific nitrogen isotopic ratios. Earth Planets Space.

[CR27] Chang Z, Vervoort JD, McClelland WC, Knaack C (2006). U–Pb dating of zircon by LA-ICP-MS. Geochem Geophys Geosyst.

[CR28] Charnley S, Rodgers S (2002). The end of interstellar chemistry as the origin of nitrogen in comets and meteorites. Astrophys J Lett.

[CR29] Citron RI, Genda H, Ida S (2015). Formation of Phobos and Deimos via a giant impact. Icarus.

[CR30] Clayton RN (1993). Oxygen isotopes in meteorites. Annu Rev Earth Planet Sci.

[CR31] Cornett DS, Reyzer ML, Chaurand P, Caprioli RM (2007). MALDI imaging mass spectrometry: molecular snapshots of biochemical systems. Nat Methods.

[CR32] Crowther SA, Gilmour JD (2012). Measuring the elemental abundance and isotopic signature of solar wind xenon collected by the Genesis mission. J Anal Spectrom.

[CR33] Day JM, Moynier F (2014). Evaporative fractionation of volatile stable isotopes and their bearing on the origin of the Moon. Phil Trans R Soc A.

[CR34] Delarue F, Robert F, Derenne S, Tartèse R, Jouvion C, Bernard S, Pont S, Gonzalez-Cano A, Duhamel R, Sugitani K (2020). Out of rock: a new look at the morphological and geochemical preservation of microfossils from the 3.46 Gyr-old Strelley Pool Formation. Precambrian Res.

[CR35] DeMeo FE, Carry B (2014). Solar system evolution from compositional mapping of the asteroid belt. Nature.

[CR36] Deng Z, Moynier F, Villeneuve J, Jensen NK, Liu D, Cartigny P, Mikouchi T, Siebert J, Agranier A, Chaussidon M, Bizzarro M (2020). Early oxidation of the Martian crust triggered by impacts. Sci Adv.

[CR37] Derenne S, Robert F (2010). Model of molecular structure of the insoluble organic matter isolated from Murchison meteorite. Meteorit Planet Sci.

[CR38] Doyle PM, Jogo K, Nagashima K, Krot AN, Wakita S, Ciesla FJ, Hutcheon ID (2015). Early aqueous activity on the ordinary and carbonaceous chondrite parent bodies recorded by fayalite. Nat Commun.

[CR39] Dreibus G, Wanke H (1985). Mars, a volatile-rich planet. Meteoritics.

[CR40] Eberhardt P, Geiss J, Graf H, Grögler N, Krähenbühl U, Schwaller H, Stettler A (1970). Trapped solar wind noble gases, Kr^81^/Kr exposure ages and K/Ar ages in Apollo 11 lunar material. Science.

[CR41] Ehlmann BL, Edwards CS (2014). Mineralogy of the Martian surface. Annu Rev Earth Planet Sci.

[CR42] Ehlmann BL, Musard JF, Murchie SL, Bibring JP, Meunier A, Fraeman AA, Langevin Y (2011). Subsurface water and clay mineral formation during the early history of Mars. Nature.

[CR43] Ehlmann BL, Anderson FS, Andrews-Hanna J, Catling DC, Christensen PR, Cohen BA, Dressing CD, Edwards CS, Elkins-Tanton LT, Farley KA, Fassett CI, Fischer WW, Fraeman AA, Golombek MP, Hamilton VE, Hayes AG, Herd CDK, Horgan B, Hu R, Jakosky BM, Johnson JR, Kasting JF, Kerber L, Kinch KM, Kite ES, Knutson HA, Lunine JI, Mahaffy PR, Mangold N, McCubbin FM, Mustard JF, Niles PB, Guantin-Nataf C, Rice MS, Stack KM, Stevenson DJ, Stewart ST, Toplis MJ, Usui T, Weiss BP, Werner SC, Wordsworth RD, Wray JJ, Yingst RA, Yung YL, Zahnle KJ (2016). The sustainability of habitability on terrestrial planets: insights, questions, and needed measurements from Mars for understanding the evolution of Earth-like worlds. J Geophys Res Planets.

[CR44] Elsila JE, Aponte JC, Blackmond DG, Burton AS, Dworkin JP, Glavin DP (2016). Meteoritic amino acids: diversity in compositions reflects parent body histories. ACS Cent Sci.

[CR45] Elsila JE, Callahan MP, Dworkin JP, Glavin DP, McLain HL, Noble SK, Gibson EK (2016). The origin of amino acids in lunar regolith samples. Geochim Cosmochim Acta.

[CR46] Fraeman AA, Arvidson RE, Murchie SL, Rivkin A, Bibring J-P, Choo TH, Gondet B, Humm D, Kuzmin RO, Manaud N, Zabalueva EV (2012). Analysis of disk-resolved OMEGA and CRISM spectral observations of Phobos and Deimos. J Geophys Res Planets.

[CR47] Fraeman AA, Murchie SL, Arvidson RE, Clark RN, Morris RV, Rivkin AS, Vilas F (2014). Spectral absorptions on Phobos and Deimos in the visible/near infrared wavelengths and their compositional constraints. Icarus.

[CR48] Friedrich JM, Wang M-S, Lipschutz ME (2002). Comparison of the trace element composition of Tagish Lake with other primitive carbonaceous chondrites. Meteorit Planet Sci.

[CR49] Friedrich JM, Glavin DP, Rivers ML, Dworkin JP (2016). Effect of a synchrotron X-ray microtomography imaging experiment on the amino acid content of a CM chondrite. Meteorit Planet Sci.

[CR50] Friedrich JM, McLain HL, Dworkin JP, Glavin DP, Towbin WH, Hill M, Ebel DS (2019). Effect of polychromatic X-ray microtomography imaging on the amino acid content of the Murchison CM chondrite. Meteorit Planet Sci.

[CR51] Fritz J, Artemieva N, Greshake A (2005). Ejection of Martian meteorites. Meteorit Planet Sci.

[CR52] Fujita K, Kurosawa K, Genda H, Hyodo R, Matsuyama S, Yamagishi A, Mikouchi T, Niihara T (2019). Assessment of the probability of microbial contamination for sample return from Martian moons I: departure of microbes from Martian surface. Life Sci Space Res.

[CR53] Fujiya W, Sugiura N, Hotta H, Ichimura K, Sano Y (2012). Evidence for the late formation of hydrous asteroids from young meteoritic carbonates. Nat Commun.

[CR54] Fujiya W, Hoppe P, Ushikubo T, Fukuda K, Lindgren P, Lee MR, Koike M, Shirai K, Sano Y (2019). Migration of D-type asteroids from the outer solar system inferred from carbonate in meteorites. Nat Astron.

[CR55] Füri E, Marty B (2015). Nitrogen isotope variations in the solar system. Nat Geosci.

[CR56] Furukawa Y, Chikaraishi Y, Ohkouchi N, Ogawa NO, Glavin DP, Dworkin JP, Abe C, Nakamura T (2019). Extraterrestrial ribose and other sugars in primitive meteorites. Proc Natl Acad Sci.

[CR57] Furusho A, Akita T, Mita M, Naraoka H, Hamase K (2020). Three-dimensional high-performance liquid chromatographic analysis of chiral amino acids in carbonaceous chondrites. J Chromatogr A.

[CR58] Genda H (2016). Origin of Earth’s oceans: an assessment of the total amount, history and supply of water. Geochem J.

[CR59] Glavin DP, Aubrey AD, Callahan MP, Dworkin JP, Elsila JE, Parker ET, Bada JL, Jenniskens P, Shaddad MH (2010). Extraterrestrial amino acids in the Almahata Sitta meteorite. Meteorit Planet Sci.

[CR60] Glavin DP, Callahan MP, Dworkin JP, Elsila JE (2011). The effects of parent body processes on amino acids in carbonaceous chondrites. Meteorit Planet Sci.

[CR61] Glavin DP, Alexander CMO’D, Aponte JC, Dworkin JP, Elsila JE, Yabuta H, Abreu NM (2018). The origin and evolution of organic matter in carbonaceous chondrites and links to their parent bodies. Primitive meteorites and asteroids.

[CR62] Grady MM, Verchovsky AB, Franchi IA, Wright IP, Pillinger CT (2002). Light dement geochemistry of the Tagish Lake CI2 chondrite: comparison with CI1 and CM2 meteorites. Meteorit Planet Sci.

[CR63] Greenwood JP, Itoh S, Sakamoto N, Vicenzi EP, Yurimoto H (2008). Hydrogen isotope evidence for loss of water from Mars through time. Geophys Res Lett.

[CR64] Greenwood RC, Barrat J-A, Miller MF, Anand M, Dauphas N, Franchi IA, Sillard P, Starkey NA (2018). Oxygen isotopic evidence for accretion of Earth’s water before a high-energy Moon-forming giant impact. Sci Adv.

[CR65] Gross JH (2006). Mass spectrometry: a textbook.

[CR66] Hamase K, Nakauchi Y, Miyoshi Y, Koga R, Kusano N, Onigahara H, Naraoka H, Mita H, Kadota Y, Nishio Y (2014). Enantioselective determination of extraterrestrial amino acids using a two-dimensional chiral high-performance liquid chromatographic system. Chromatography.

[CR67] Harada K, Hare P, Windsor C, Fox S (1971). Evidence for compounds hydrolyzable to amino acids in aqueous extracts of Apollo 11 and Apollo 12 lunar fines. Science.

[CR68] Hare P, Harada K, Fox S (1970) Analyses for amino acids in lunar fines. In: Proceedings of the Apollo 11 lunar science conference, Geochimica et Cosmochimica Acta Supplement, vol 1, pp 1799–1803

[CR69] Hartmann WK (1990). Additional evidence about an early intense flux of C asteroids and the origin of Phobos. Icarus.

[CR70] Hashiguchi M, Kobayashi S, Yurimoto H (2015). Deuterium- and ^15^N-signatures of organic globules in Murchison and Northwest Africa 801 meteorites. Geochem J.

[CR71] Herd CDK, Blinova A, Simkus DN, Huang Y, Tarozo R, Alexander CMO’D, Gyngard F, Nittler LR, Cody GD, Fogel ML, Kebukawa Y, Kilcoyne ALD, Hilts RW, Slater GF, Glavin DP, Dworkin JP, Callahan MP, Elsila JE, De Gregorio BT, Stroud RM (2011). Origin and evolution of prebiotic organic matter as inferred from the Tagish Lake meteorite. Science.

[CR72] Hertkorn N, Harir M, Schmitt-Kopplin P (2015). Nontarget analysis of Murchison soluble organic matter by high-field NMR spectroscopy and FTICR mass spectrometry. Magn Reson Chem.

[CR73] Hertzog J, Naraoka H, Schmitt-Kopplin P (2019). Profiling Murchison soluble organic matter for new organic compounds with APPI-and ESI-FT-ICR MS. Life.

[CR74] Herwartz D, Pack A, Friedrichs B, Bischoff A (2014). Identification of the giant impactor Theia in lunar rocks. Science.

[CR75] Hibiya Y, Iizuka T, Yamashita K, Yoneda S, Yamakawa A (2019). Sequential chemical separation of Cr and Ti from a single digest for high-precision isotope measurements of planetary materials. Geostand Geoanal Res.

[CR76] Higuchi A, Ida S (2017). Temporary capture of asteroids by an eccentric planet. Astron J.

[CR77] Hiroi T, Zolensky ME, Pieters CM (2001). The Tagish Lake meteorite: a possible sample from a D-type asteroid. Science.

[CR78] Hiroi T, Tonui E, Pieters CM, Zolensky ME, Ueda Y, Miyamoto M, Sasaki S (2005) Meteorite WIS 91600: a new sample related to a D-or T-type asteroid. In: 36th lunar and planetary science conference, vol 36, p 1564

[CR79] Hoppe P (2006). NanoSIMS: a new tool in cosmochemistry. Appl Surf Sci.

[CR80] Hoppe P, Leitner J, Kodolányi J (2017). The stardust abundance in the local interstellar cloud at the birth of the solar system. Nat Astron.

[CR81] Huang Y, Alexandre MR, Wang Y (2007). Structure and isotopic ratios of aliphatic side chains in the insoluble organic matter of the Murchison carbonaceous chondrite. Earth Planet Sci Lett.

[CR82] Hyodo R, Genda H, Charnoz S, Rosenblatt P (2017). On the impact origin of Phobos and Deimos. I. Thermodynamic and physical aspects. Astrophys J.

[CR83] Hyodo R, Kurosawa K, Genda H, Usui T, Fujita K (2019). Transport of impact ejecta from Mars to its moons as a means to reveal Martian history. Sci Rep.

[CR84] Ito M, Uesugi M, Naraoka H, Yabuta H, Kitajima F, Mita H, Takano Y, Karouji Y, Yada T, Ishibashi Y (2014). H, C, and N isotopic compositions of Hayabusa category 3 organic samples. Earth Planets Space.

[CR85] Jakosky BM (2021). Atmospheric loss to space and the history of water on Mars. Annu Rev Earth Planet Sci.

[CR86] Jourdan F, Timms NE, Eroglu E, Mayers C, Frew A, Bland P, Collins GS, Davison TM, Abe M, Yada T (2017). Collisional history of asteroid Itokawa. Geology.

[CR87] Joy KH, Crawford IA, Downes H, Russell SS, Kearsley AT (2006). A petrological, mineralogical, and chemical analysis of the lunar mare basalt meteorite LaPaz Icefield 02205, 02224, and 02226. Meteorit Planet Sci.

[CR88] Kanno A, Hiroi T, Nakamura R, Abe M, Ishiguro M, Hasegawa S, Miyasaka S, Sekiguchi T, Terada H, Igarashi G (2003). The first detection of water absorption on a D type asteroid. Geophys Res Lett.

[CR89] Karner J, Papike JJ, Shearer CK (2003). Olivine from planetary basalts: chemical signatures that indicate planetary parentage and those that record igneous setting and process. Am Miner.

[CR90] Karner J, Papike JJ, Shearer CK (2006). Comparative planetary mineralogy: pyroxene major- and minor-element chemistry and partitioning of vanadium between pyroxene and melt in planetary basalts. Am Miner.

[CR91] Kato C, Moynier F (2017). Gallium isotopic evidence for extensive volatile loss from the Moon during its formation. Sci Adv.

[CR92] Kato C, Moynier F, Foriel J, Teng F-Z, Puchtel IS (2017). The gallium isotopic composition of the bulk silicate Earth. Chem Geol.

[CR93] Kato H, Satou Y, Yoshikawa K, Otsuki M, Sawada H, Kuratomi T, Hidaka N (2020) Subsurface sampling robot for time-limited asteroid exploration. In: Proceedings of IEEE/RSJ international conference on intelligent robots and systems (IROS), pp 1925–1932

[CR94] Kawasaki N, Park C, Sakamoto N, Park SY, Kim HN, Kuroda M, Yurimoto H (2019). Variations in initial ^26^Al/^27^Al ratios among fluffy type A Ca–Al-rich inclusions from reduced CV chondrites. Earth Planet Sci Lett.

[CR95] Kebukawa Y, Okudaira K, Yabuta H, Hasegawa S, Tabata M, Furukawa Y, Ito M, Nakato A, Kilcoyne ALD, Kobayashi K, Yokobori S, Imai E, Kawaguchi Y, Yano H, Yamagishi A (2019). STXM-XANES analyses of Murchison meteorite samples captured by aerogel after hypervelocity impacts: a potential implication of organic matter degradation for micrometeoroid collection experiments. Geochem J.

[CR96] Kebukawa Y, Zolensky ME, Ito M, Ogawa NO, Takano Y, Ohkouchi N, Nakato A, Suga H, Takeichi Y, Takahashi Y, Kobayashi K (2020). Primordial organic matter in the xenolithic clast in the Zag H chondrite: possible relation to D/P asteroids. Geochim Cosmochim Acta.

[CR97] Kitajima F, Uesugi M, Karouji Y, Ishibashi Y, Yada T, Naraoka H, Abe M, Fujimura A, Ito M, Yabuta H, Mita H, Takano Y, Okada T (2015). A micro-Raman and infrared study of the several Hayabusa category 3 (organic) particles. Earth Planets Space.

[CR98] Koike M, Sugiura N, Takahata N, Ishida A, Sano Y (2017). U–Pb and Hf-W dating of young zircon in mesosiderite Asuka 882023. Geophys Res Lett.

[CR99] Koike M, Nakada R, Kajitani I, Usui T, Tamenori Y, Sugahara H, Kobayashi A (2020). In-situ preservation of nitrogen-bearing organics in Noachian Martian carbonates. Nat Commun.

[CR100] Krot AN, Nagashima K, Alexander CMO’D, Ciesla FJ, Fujiya W, Bonal L, Michel P, DeMeo FE, Bottke WF (2015). Sources of water and aqueous activity on the chondrite parent asteroids. Asteroids IV.

[CR101] Kruijer TS, Burkhardt C, Budde G, Kleine T (2017). Age of Jupiter inferred from the distinct genetics and formation times of meteorites. Proc Natl Acad Sci.

[CR102] Kruijer TS, Kleine T, Borg LE (2019). The great isotopic dichotomy of the early solar system. Nat Astron.

[CR103] Kuramoto K, Kawakatsu Y, Fujimoto M, Genda H, Imamura T, Kameda S, Matsumoto K, Miyamoto H, Morota T, Nagaoka H, Nakamura T, Ogawa K, Otake H, Ozaki M, Sasaki S, Senshu H, Tachibana S, Terada N, Usui T, Wada K, Watanabe S, MMX Study Team (2018) Martian Moons eXploration (MMX) conceptual study update. In: Lunar and planetary science conference, vol 49, p 2143

[CR104] Kurokawa H, Sato M, Ushioda M, Matsuyama T, Moriwaki R, Dohm JM, Usui T (2015). Evolution of water reservoirs on Mars: constraints from hydrogen isotopes in Martian meteorites. Earth Planet Sci Lett.

[CR105] Kurosawa K, Okamoto T, Genda H (2018). Hydrocode modeling of the spallation process during hypervelocity impacts: implications for the ejection of Martian meteorites. Icarus.

[CR106] Kurosawa K, Genda H, Hyodo R, Yamagishi A, Mikouchi T, Niihara T, Matsuyama S, Fujita K (2019). Assessment of the probability of microbial contamination for sample return from Martian moons II: the fate of microbes on Martian moons. Life Sc Space Res.

[CR107] Lawrence DJ, Peplowski PN, Beck AW, Burks MT, Chabot NL, Cully MJ, Elphic RC, Ernst CM, Fix S, Goldsten JO, Hoffer EM, Kusano H, Murchie SL, Schratz BC, Usui T, Yokley ZW (2019). Measuring the elemental composition of phobos: the Mars–moon exploration with gamma rays and neutrons (MEGANE) investigation for the Martian moons exploration (MMX) mission. Earth Space Sci.

[CR108] Leitner J, Metzler K, Vollmer C, Floss C, Haenecour P, Kodolányi J, Harries D, Hoppe P (2020). The presolar grain inventory of fine-grained chondrule rims in the Mighei-type (CM) chondrites. Meteorit Planet Sci.

[CR109] Leroux H, Cuvillier P, Zanda B, Hewins RH (2015). GEMS-like material in the matrix of the Paris meteorite and the early stages of alteration of CM chondrites. Geochim Cosmochim Acta.

[CR110] Lewis KW, Aharonson O, Grotzinger JP, Kirk RL, McEwen AS, Suer T-A (2008). Quasi-periodic bedding in the sedimentary rock record of Mars. Science.

[CR111] Lugmair GW, Shukolyukov A (1998). Early solar system timescales according to ^53^Mn–^53^Cr systematics. Geochim Cosmochim Acta.

[CR112] Malin MC, Edgett KS (2000). Sedimentary rocks of early Mars. Science.

[CR113] Marty B (2012). The origins and concentrations of water, carbon, nitrogen and noble gases on Earth. Earth Planet Sci Lett.

[CR114] Marty B, Avice G, Sano Y, Altwegg K, Balsiger H, Hässig M, Morbidelli A, Mousis O, Rubin M (2016). Origins of volatile elements (H, C, N, noble gases) on Earth and Mars in light of recent results from the ROSETTA cometary mission. Earth Planet Sci Lett.

[CR115] Matsumoto T, Tsuchiyama A, Miyake A, Noguchi T, Nakamura M, Uesugi K, Takeuchi A, Suzuki Y, Nakano T (2015). Surface and internal structures of a space-weathered rim of an Itokawa regolith particle. Icarus.

[CR116] Mazor E, Heymann D, Anders E (1970). Noble gases in carbonaceous chondrites. Geochim Cosmochim Acta.

[CR117] McCubbin FM, Herd CDK, Yada T, Hutzler A, Calaway MJ, Allton JH, Corrigan CM, Fries MD, Harrington AD, McCoy TJ (2019). Advanced curation of astromaterials for planetary science. Space Sci Rev.

[CR118] McDonnell LA, Heeren R (2007). Imaging mass spectrometry. Mass Spectrom Rev.

[CR119] McNair HM, Miller JM, Snow NH (2019). Basic gas chromatography.

[CR120] McSween HY (2015). Petrology of Mars. Am Miner.

[CR121] Meshik A, Hohenberg C, Pravdivtseva O, Burnett D (2014). Heavy noble gases in solar wind delivered by Genesis mission. Geochim Cosmochim Acta.

[CR122] Mimura K, Okamoto M, Sugitani K, Hashimoto S (2007). Selective release of D and ^13^C from insoluble organic matter of the Murchison meteorite by impact shock. Meteorit Planet Sci.

[CR123] Monkawa A, Mikouchi T, Koizumi E, Sugiyama K, Miyamoto M (2006). Determination of the Fe oxidation state of the Chassigny kaersutite: a microXANES spectroscopic study. Meteorit Planet Sci.

[CR124] Montgomery W, Bromiley GD, Sephton MA (2016). The nature of organic records in impact excavated rocks on Mars. Sci Rep.

[CR125] Mougel B, Moynier F, Göpel C (2018). Chromium isotopic homogeneity between the Moon, the Earth, and enstatite chondrites. Earth Planet Sci Lett.

[CR126] Moynier F, Hu Y, Wang K, Zhao Y, Gérard Y, Deng Z, Moureau J, Li W, Simon JI, Teng F-Z (2021). Potassium isotopic composition of various samples using a dual-path collision cell-capable multiple-collector inductively coupled plasma mass spectrometer, Nu instruments Sapphire. Chem Geol.

[CR127] Murchie S, Erard S (1996). Spectral properties and heterogeneity of Phobos from measurements by Phobos 2. Icarus.

[CR128] Nagao K, Okazaki R, Nakamura T, Miura YN, Osawa T, Bajo K, Matsuda S, Ebihara M, Ireland TR, Kitajima F, Naraoka H, Noguchi T, Tsuchiyama A, Yurimoto H, Zolensky ME, Uesugi M, Shirai K, Abe M, Yada T, Ishibashi Y, Fujimura A, Mukai T, Ueno M, Okada T, Yoshikawa M, Kawaguchi J (2011). Irradiation history of Itokawa regolith material deduced from noble gases in the Hayabusa samples. Science.

[CR129] Nakamura T, Noguchi T, Tanaka M, Zolensky ME, Kimura M, Tsuchiyama A, Nakato A, Ogami T, Ishida H, Uesugi M, Yada T, Shirai K, Fujimura A, Okazaki R, Sandford SA, Ishibashi Y, Abe M, Okada T, Ueno M, Mukai T, Yoshikawa M, Kawaguchi J (2011). Itokawa dust particles: a direct link between S-type asteroids and ordinary chondrites. Science.

[CR130] Nakamura E, Makishima A, Moriguti T, Kobayashi K, Tanaka R, Kunihiro T, Tsujimori T, Sakaguchi C, Kitagawa H, Ota T, Yachi Y, Yada T, Abe M, Fujimura A, Ueno M, Mukai T, Yoshikawa M, Kawaguchi J (2012). Space environment of an asteroid preserved on micrograins returned by the Hayabusa spacecraft. Proc Natl Acad Sci.

[CR131] Nakamura-Messenger K, Messenger S, Keller LP, Clemett SJ, Zolensky ME (2006). Organic globules in the Tagish Lake meteorite: remnants of the protosolar disk. Science.

[CR132] Nakashima D, Kita NT, Ushikubo T, Noguchi T, Nakamura T, Valley JW (2013). Oxygen three-isotope ratios of silicate particles returned from asteroid Itokawa by the Hayabusa spacecraft: a strong link with equilibrated LL chondrites. Earth Planet Sci Lett.

[CR133] Nakashima D, Ushikubo T, Kita NT, Weisberg MK, Zolensky ME, Ebel DS (2015). Late formation of a comet Wild 2 crystalline silicate particle, Pyxie, inferred from Al–Mg chronology of plagioclase. Earth Planet Sci Lett.

[CR134] Naraoka H, Hashiguchi M (2018). In situ organic compound analysis on a meteorite surface by desorption electrospray ionization coupled with an Orbitrap mass spectrometer. Rapid Commun Mass Spectrom.

[CR135] Naraoka H, Hashiguchi M (2019). Distinct distribution of soluble N-heterocyclic compounds between CM and CR chondrites. Geochem J.

[CR136] Naraoka H, Mita H, Hamase K, Mita M, Yabuta H, Saito K, Fukushima K, Kitajima F, Sandford SA, Nakamura T, Noguchi T, Okazaki R, Nagao K, Ebihara M, Yurimoto H, Tsuchiyama A, Abe M, Shirai K, Ueno M, Yada T, Ishibashi Y, Okada T, Fujimura A, Mukai T, Yoshikawa M, Kawaguchi J (2012). Preliminary organic compound analysis of microparticles returned from Asteroid 25143 Itokawa by the Hayabusa mission. Geochem J.

[CR137] Naraoka H, Aoki D, Fukushima K, Uesugi M, Ito M, Kitajima F, Mita H, Yabuta H, Takano Y, Yada T, Ishibashi Y, Karouji Y, Okada T, Abe M (2015). ToF-SIMS analysis of carbonaceous particles in the sample catcher of the Hayabusa spacecraft. Earth Planets Space.

[CR138] Naraoka H, Yamashita Y, Yamaguchi M, Orthous-Daunay F-R (2017). Molecular evolution of N-containing cyclic compounds in the parent body of the Murchison meteorite. ACS Earth Space Chem.

[CR139] Naraoka H, Hashiguchi M, Sato Y, Hamase K (2019). New applications of high-resolution analytical methods to study trace organic compounds in extraterrestrial materials. Life.

[CR160] National Academies of Sciences, Engineering, and Medicine (2019). Planetary protection classification of sample return missions from the Martian moons.

[CR140] Nayak M, Nimmo F, Udrea B (2016). Effects of mass transfer between Martian satellites on surface geology. Icarus.

[CR141] Noguchi T, Nakamura T, Kimura M, Zolensky ME, Tanaka M, Hashimoto T, Konno M, Nakato A, Ogami T, Fujimura A, Abe M, Yada T, Mukai T, Ueno M, Okada T, Shirai K, Ishibashi Y, Okazaki R (2011). Incipient space weathering observed on the surface of Itokawa dust particles. Science.

[CR142] Noguchi T, Yabuta H, Itoh S, Sakamoto N, Mitsunari T, Okubo A, Okazaki R, Nakamura T, Tachibana S, Terada K, Ebihara M, Imae N, Kimura M, Nagahara H (2017). Variation of mineralogy and organic material during the early stages of aqueous activity recorded in Antarctic micrometeorites. Geochim Cosmochim Acta.

[CR143] Nyquist LE, Bogard DD, Shih C-Y, Greshake A, Stöffler D, Eugster O, Kallenbach R, Geiss J, Hartmann WK (2001). Ages and geologic histories of Martian meteorites. Chronology and evolution of Mars.

[CR144] Oba Y, Takano Y, Naraoka H, Watanabe N, Kouchi A (2019). Nucleobase synthesis in interstellar ices. Nat Commun.

[CR145] Oba Y, Takano Y, Naraoka H, Furukawa Y, Glavin DP, Dworkin JP, Tachibana S (2020). Extraterrestrial hexamethylenetetramine in meteorites-a precursor of prebiotic chemistry in the inner solar system. Nat Commun.

[CR146] Ogawa ON, Nagata T, Kitazato H, Ohkouchi N, Ohkouchi N, Tayasu I, Koba K (2010). Ultra-sensitive elemental analyzer/isotope ratio mass spectrometer for stable nitrogen and carbon isotope analyses. Earth, life and isotopes.

[CR147] Ogawa N, Takano Y, Ohkouchi N, Hashiguchi M, Parker E, Mclain HL, Dworkin JP, Naraoka H (2020) Quantification and isotopic measurements of submicrogram scales Carbon and Nitrogen from extraterrestrial materials through nano-EA/IRMS. In: Lunar and planetary science conference, vol 51, p 1926

[CR148] Okazaki R, Sawada H, Yamanouchi S, Tachibana S, Miura Y, Sakamoto K, Takano Y, Abe M, Itoh S, Yamada K, Yabuta H, Okamoto C, Yano H, Noguchi T, Nakamura T, Nagao K (2017). Hayabusa2 sample container: metal-seal system for vacuum encapsulation of returned samples. Space Sci Rev.

[CR149] Orthous-Daunay F-R, Quirico E, Lemelle L, Beck P, deAndrade V, Simionovici A, Derenne S (2010). Speciation of sulfur in the insoluble organic matter from carbonaceous chondrites by XANES spectroscopy. Earth Planet Sci Lett.

[CR150] Orthous-Daunay F-R, Wolters C, Flandinet L, Vuitton V, Beck P, Bonal L, Isa J, Moynier F, Voisin D, Moran S (2019) Comparison of molecular complexity between chondrites, Martian meteorite and lunar soils. In: Annual meeting of the Meteoritical Society, vol 82, p 2157

[CR151] Orthous-Daunay F-R, Wolters C, Vuitton V, Isa J, Naraoka H, Thissen R (2020) Orbitrap-MS and chromatography in preparation for Hayabusa2 molecular complexity analyses. In: Lunar and planetary science conference, vol 51, p 2551

[CR152] Pajola M, Lazzarin M, Dalle Ore CM, Cruikshank DP, Roush TL, Magrin S, Bertini I, La Forgia F, Barbieri C (2013). Phobos as a D-type captured asteroid, spectral modeling from 0.25 to 4.0 μm. Astrophys J.

[CR153] Paniello RC, Day JMD, Moynier F (2012). Zinc isotopic evidence for the origin of the Moon. Nature.

[CR154] Pant NC, Jimenez-Espejo JF, Cook CP, Biswas P, Upadhyay D, Kuroda J, Ito M, Macky R, Marchesi C, Shimizu K, Senda R, van de Flierdt T, Takano Y, Suzuki K, Escutia C, Shrivastava PK (2018). Suspected meteorite fragments in marine sediments from East Antarctica. Antarct Sci.

[CR155] Papike JJ, Karner JM, Shearer CK, Burger PV (2009). Silicate mineralogy of martian meteorites. Geochim Cosmochim Acta.

[CR156] Park J, Turrin BD, Herzog GF, Lindsay FN, Delaney JS, Swisher CC, Uesugi M, Karouji Y, Yada T, Abe M, Okada T, Ishibashi Y (2015). ^40^Ar/^39^Ar age of material returned from asteroid 25143 Itokawa. Meteorit Planet Sci.

[CR157] Pätzold M, Andert T, Jacobson R, Rosenblatt P, Dehant V (2014). Phobos: observed bulk properties. Planet Space Sci.

[CR158] Pignatale FC, Charnoz S, Rosenblatt P, Hyodo R, Nakamura T, Genda H (2018). On the impact origin of Phobos and Deimos. III. Resulting composition from different impactors. Astrophys J.

[CR159] Pizzarello S, Huang YS, Becker L, Poreda RJ, Nieman RA, Cooper G, Williams M (2001). The organic content of the Tagish Lake meteorite. Science.

[CR161] Pringle EA, Moynier F (2017). Rubidium isotopic composition of the Earth, meteorites, and the Moon: evidence for the origin of volatile loss during planetary accretion. Earth Planet Sci Lett.

[CR162] Pringle EA, Moynier F, Beck P, Paniello R, Hezel DC (2017). The origin of volatile element depletion in early solar system material: clues from Zn isotopes in chondrules. Earth Planet Sci Lett.

[CR163] Quirico E, Moroz LV, Schmitt B, Arnold G, Faure M, Beck P, Bonal L, Ciarniello M, Capaccioni F, Filacchione G, Erard S, Leyrat C, Bockelée-Morvan D, Zinzi A, Palomba E, Drossart P, Tosi F, Capria MT, De Sanctis MC, Raponi A, Fonti S, Mancarella F, Orofino V, Barucci A, Blecka MI, Carlson R, Despan D, Faure A, Fornasier S, Gudipati MS, Longobardo A, Markus K, Mennella V, Merlin F, Piccioni G, Rousseau B, Taylor F (2016). Refractory and semi-volatile organics at the surface of comet 67P/Churyumov-Gerasimenko: insights from the VIRTIS/Rosetta imaging spectrometer. Icarus.

[CR164] Ramsley KR, Head JW (2013). Mars impact ejecta in the regolith of Phobos: bulk concentration and distribution. Planet Space Sci.

[CR165] Righter K, Chabot NL (2011). Moderately and slightly siderophile element constraints on the depth and extent of melting in early Mars. Meteorit Planet Sci.

[CR166] Rivkin AS, Brown RH, Trilling DE, Bell JF, Plassmann JH (2002). Near-infrared spectrophotometry of Phobos and Deimos. Icarus.

[CR167] Rivkin AS, Howell ES, Vilas F, Lebofsky LA, Bottke WF, Cellino A, Paolicchi P, Bonzel RP (2002). Hydrated minerals on asteroids: the astronomical record. Asteroids III.

[CR168] Rochette P, Lorand J-P, Fillion G, Sautter V (2001). Pyrrhotite and the remanent magnetization of SNC meteorites: a changing perspective on Martian magnetism. Earth Planet Sci Lett.

[CR169] Rosenblatt P (2011). The origin of the Martian moons revisited. Astron Astrophys Rev.

[CR170] Rosenblatt P, Charnoz S, Dunseath KM, Terao-Dunseath M, Trinh A, Hyodo R, Genda H, Toupin S (2016). Accretion of Phobos and Deimos in an extended debris disc stirred by transient moons. Nat Geosci.

[CR171] Sawada H, Okazaki R, Tachibana S, Sakamoto K, Takano Y, Okamoto C, Yano H, Miura Y, Abe M, Hasegawa S, Noguchi T, Hayabusa2 Sampler Team (2017). Hayabusa2 sampler: collection of asteroidal surface material. Space Sci Rev.

[CR172] Scherer J, Paul J, O'keefe A, Saykally R (1997). Cavity ringdown laser absorption spectroscopy: history, development, and application to pulsed molecular beams. Chem Rev.

[CR173] Schiller M, Bizzarro M, Fernandes VA (2018). Isotopic evolution of the protoplanetary disk and the building blocks of Earth and the Moon. Nature.

[CR174] Schmedemann N, Michael GG, Ivanov BA, Murray JB, Neukum G (2014). The age of Phobos and its largest crater, Stickney. Planet Space Sci.

[CR175] Schmitt-Kopplin P, Gabelica Z, Gougeon RD, Fekete A, Kanawati B, Harir M, Gebefuegi I, Eckel G, Hertkorn N (2010). High molecular diversity of extraterrestrial organic matter in Murchison meteorite revealed 40 years after its fall. Proc Natl Acad Sci.

[CR176] Scott ERD, Krot AN, Davis AM (2003). Chondrites and their components. Meteorites, comets and planets. Treatise on geochemistry.

[CR177] Sephton MA, Verchovsky AB, Bland PA, Gilmour I, Grady MM, Wright IP (2003). Investigating the variations in carbon and nitrogen isotopes in carbonaceous chondrites. Geochim Cosmochim Acta.

[CR178] Smith KE, Callahan MP, Gerakines PA, Dworkin JP, House CH (2014). Investigation of pyridine carboxylic acids in CM2 carbonaceous chondrites: potential precursor molecules for ancient coenzymes. Geochim Cosmochim Acta.

[CR179] Snyder LR, Kirkland JJ, Dolan JW (2011). Introduction to modern liquid chromatography.

[CR180] Sossi PA, Nebel O, O'Neill HSC, Moynier F (2018). Zinc isotope composition of the Earth and its behaviour during planetary accretion. Chem Geol.

[CR181] Srinivasan G, Whitehouse MJ, Weber I, Yamaguchi A (2007). The crystallization age of eucrite zircon. Science.

[CR182] Stead CV, Tomlinson EL, Kamber BS, Babechuk MG, McKenna CA (2017). Rare earth element determination in olivine by laser ablation-quadrupole-ICP-MS: an analytical strategy and applications. Geostand Geoanal Res.

[CR183] Sugahara H, Mimura K (2014). Glycine oligomerization up to triglycine by shock experiments simulating comet impacts. Geochem J.

[CR184] Sutton SR, Lanzirotti A, Newville M, Dyar MD, Delaney J (2020). Oxybarometry and valence quantification based on microscale X-ray absorption fine structure (XAFS) spectroscopy of multivalent elements. Chem Geol.

[CR185] Tachibana S, Abe M, Arakawa M, Fujimoto M, Iijima Y, Ishiguro M, Kitazato K, Kobayashi N, Namiki N, Okada T (2014). Hayabusa2: scientific importance of samples returned from C-type near-Earth asteroid (162173) 1999 JU3. Geochem J.

[CR186] Takano Y, Chikaraishi Y, Ohkouchi N (2015). Isolation of underivatized amino acids by ion-pair high performance liquid chromatography for precise measurement of nitrogen isotopic composition of amino acids: development of comprehensive LC x GC/C/IRMS method. Int J Mass Spectrom.

[CR187] Takano Y, Yamada K, Okamoto C, Sawada H, Okazaki R, Sakamoto K, Kebukawa Y, Kiryu K, Shibuya T, Igisu M, Yano H, Tachibana S, Hayabusa2 Project Team (2020). Chemical assessment of the explosive chamber in the projector system of Hayabusa2 for asteroid sampling. Earth Planets Space.

[CR188] Takáts Z, Wiseman JM, Gologan B, Cooks RG (2004). Mass spectrometry sampling under ambient conditions with desorption electrospray ionization. Science.

[CR189] Taylor SR (1975). Lunar science: a post-Apollo view: scientific results and insights from the lunar samples.

[CR190] Taylor LA, Nazarov MA, Shearer CK, McSween HY, Cahill J, Neal CR, Ivanova MA, Barsukova LD, Lentz RC, Clayton RN, Mayeda TK (2002). Martian meteorite Dhofar 019: a new shergottite. Meteorit Planet Sci.

[CR191] Terada K, Sano Y, Takahata N, Ishida A, Tsuchiyama A, Nakamura T, Noguchi T, Karouji Y, Uesugi M, Yada T, Nakabayashi M, Fukuda K, Nagahara H (2018). Thermal and impact histories of 25143 Itokawa recorded in Hayabusa particles. Sci Rep.

[CR192] Tittel FK, Richter D, Fried A, Sorokina IT, Vodopyanov KL (2003). Mid-infrared laser applications in spectroscopy. Solid-state mid-infrared laser sources.

[CR193] Tomeoka K, Buseck PR (1988). Matrix mineralogy of the Orgueil CI carbonaceous chondrite. Geochim Cosmochim Acta.

[CR194] Trieloff M, Jessberger EK, Herrwerth I, Hopp J, Fiéni C, Ghélis M, Bourot-Denise M, Pellas P (2003). Structure and thermal history of the H-chondrite parent asteroid revealed by thermochronometry. Nature.

[CR195] Trinquier A, Birck J-L, Allègre CJ (2007). Widespread ^54^Cr heterogeneity in the inner solar system. Astrophys J.

[CR196] Trinquier A, Birck J-L, Allègre CJ, Göpel C, Ulfbeck D (2008). ^53^Mn-^53^Cr systematics of the early solar system revisited. Geochim Cosmochim Acta.

[CR197] Trinquier A, Elliott T, Ulfbeck D, Coath C, Krot AN, Bizzarro M (2009). Origin of nucleosynthetic isotope heterogeneity in the solar protoplanetary disk. Science.

[CR198] Tsuchiyama A, Uesugi M, Matsushima T, Michikami T, Kadono T, Nakamura T, Uesugi K, Nakano T, Sandford SA, Noguchi R, Matsumoto T, Matsuno J, Nagano T, Imai Y, Takeuchi A, Suzuki Y, Ogami T, Katagiri J, Ebihara M, Ireland TR, Kitajima F, Nagao K, Naraoka H, Noguchi T, Okazaki R, Yurimoto H, Zolensky ME, Mukai T, Abe M, Yada T, Fujimura A, Yoshikawa M, Kawaguchi J (2011). Three-dimensional structure of Hayabusa samples: origin and evolution of Itokawa regolith. Science.

[CR199] Uesugi M, Naraoka H, Ito M, Yabuta H, Kitajima F, Takano Y, Mita H, Ohnishi I, Kebukawa Y, Yada T, Karouji Y, Ishibashi Y, Okada T, Abe M (2014). Sequential analysis of carbonaceous materials in Hayabusa-returned samples for the determination of their origin. Earth Planets Space.

[CR200] Uesugi M, Ito M, Yabuta H, Naraoka H, Kitajima F, Takano Y, Mita H, Kebukawa Y, Nakato A, Karouji Y (2019). Further characterization of carbonaceous materials in Hayabusa-returned samples to understand their origin. Meteorit Planet Sci.

[CR201] Usui T, Yamagishi A, Kakegawa T, Usui T (2019). What geology and mineralogy tell us about water on Mars. Astrobiology.

[CR202] Usui T, Alexander CMO’D, Wang J, Simon JI, Jones JH (2012). Origin of water and mantle–crust interactions on Mars inferred from hydrogen isotopes and volatile element abundances of olivine-hosted melt inclusions of primitive shergottites. Earth Planet Sci Lett.

[CR203] Usui T, Alexander CMO’D, Wang J, Simon JI, Jones JH (2015). Meteoritic evidence for a previously unrecognized hydrogen reservoir on Mars. Earth Planet Sci Lett.

[CR204] Usui T, Bajo K, Fujiya W, Furukawa Y, Koike M, Miura YN, Sugahara H, Tachibana S, Takano Y, Kuramoto K (2020). The importance of Phobos sample return for understanding the Mars–moon system. Space Sci Rev.

[CR205] van Kooten E, Moynier F (2019). Zinc isotope analyses of singularly small samples (< 5 ng Zn): investigating chondrule-matrix complementarity in Leoville. Geochim Cosmochim Acta.

[CR206] Vokrouhlický D, Bottke WF, Nesvorný D (2016). Capture of trans-Neptunian planetesimals in the main asteroid belt. Astron J.

[CR207] Walker RJ (2009). Highly siderophile elements in the Earth, Moon and Mars: update and implications for planetary accretion and differentiation. Geochemistry.

[CR208] Warren PH (2011). Stable-isotopic anomalies and the accretionary assemblage of the Earth and Mars: a subordinate role for carbonaceous chondrites. Earth Planet Sci Lett.

[CR209] Watrous JD, Dorrestein PC (2011). Imaging mass spectrometry in microbiology. Nat Rev Microbiol.

[CR210] Weisberg MK, Prinz M, Clayton RN, Mayeda TK (1993). The CR (Renazzo-type) carbonaceous chondrite group and its implications. Geochim Cosmochim Acta.

[CR211] Wieler R, Kehm K, Meshik AP, Hohenberg CM (1996). Secular changes in the xenon and krypton abundances in the solar wind recorded in single lunar grains. Nature.

[CR212] Yabuta H, Uesugi M, Naraoka H, Ito M, Kilcoyne ALD, Sandford SA, Kitajima F, Mita H, Takano Y, Yada T, Karouji Y, Ishibashi Y, Okada T, Abe M (2014). X-ray absorption near edge structure spectroscopic study of Hayabusa category 3 carbonaceous particles. Earth Planets Space.

[CR213] Yamamoto S, Watanabe S, Matsunaga T (2018). Space-weathered anorthosite as spectral D-type material on the Martian satellites. Geophys Res Lett.

[CR214] Yokoyama TD, Suzuki T, Kon Y, Hirata T (2011). Determinations of rare earth element abundance and U–Pb age of zircons using multispot laser ablation-inductively coupled plasma mass spectrometry. Anal Chem.

[CR215] Yoshimura T, Takano Y, Hashiguchi M, Ogawa N, Ohkouchi N, Dworkin JP, Naraoka H (2020) Major and trace element composition in acid-soluble extracts of Murchison and Yamato meteorites. In: Lunar and planetary science conference, vol 51, p 1971

[CR216] Young ED, Simon JI, Galy A, Russell SS, Tonui E, Lovera O (2005). Supra-canonical ^26^Al/^27^Al and the residence time of CAIs in the solar protoplanetary disk. Science.

[CR217] Young ED, Kohl IE, Warren PH, Rubie DC, Jacobson SA, Morbidelli A (2016). Oxygen isotopic evidence for vigorous mixing during the Moon-forming giant impact. Science.

[CR218] Yurimoto H, Abe K, Abe M, Ebihara M, Fujimura A, Hashiguchi M, Hashizume K, Ireland TR, Itoh S, Katayama J, Kato C, Kawaguchi J, Kawasaki N, Kitajima F, Kobayashi S, Meike T, Mukai T, Nagao K, Nakamura T, Naraoka H, Noguchi T, Okazaki R, Park C, Sakamoto N, Seto Y, Takei M, Tsuchiyama A, Uesugi M, Wakaki S, Yada T, Yamamoto K, Yoshikawa M, Zolensky ME (2011). Oxygen isotopic compositions of asteroidal materials returned from Itokawa by the Hayabusa mission. Science.

[CR219] Zacny K, Thomas L, Paulsen G, Van Dyne D, Lam S, Williams H, Sabahi D, Ng P, Satou Y, Kato H, Sawada H, Usui T, Fujimoto M, Mueller R, Zolensky M, Statler T, Dudzinsky L, Chu P (2020) Pneumatic sampler (P-sampler) for the Martian Moons eXploration (MMX) Mission. In: 2020 IEEE aerospace conference, pp 1–11

[CR220] Zhang J, Dauphas N, Davis AM, Leya I, Fedkin A (2012). The proto-Earth as a significant source of lunar material. Nat Geosci.

[CR221] Zhu K, Moynier F, Schiller M, Wielandt D, Larsen KK, van Kooten EMME, Barrat J-A, Bizzarro M (2020). Chromium isotopic constraints on the origin of the ureilite parent body. Astrophys J.

[CR222] Zolensky ME, Nakamura K, Gounelle M, Mikouchi T, Kasama T, Tachikawa O, Tonui E (2002). Mineralogy of Tagish Lake: an ungrouped type 2 carbonaceous chondrite. Meteorit Planet Sci.

